# Incidence of Severe Malaria Syndromes and Status of Immune Responses among Khat Chewer Malaria Patients in Ethiopia

**DOI:** 10.1371/journal.pone.0131212

**Published:** 2015-07-14

**Authors:** Tsige Ketema, Ketema Bacha, Esayas Alemayehu, Argaw Ambelu

**Affiliations:** 1 Department of Environmental Health Sciences and Technology, College of Health Sciences, Jimma University, Jimma, Ethiopia; 2 Department of Biology,College of Natural Sciences, Jimma University, Jimma, Ethiopia; 3 School of Civil and Environmental Engineering, Institute of Technology, Jimma University, Jimma, Ethiopia; Centro de Pesquisa Rene Rachou/Fundação Oswaldo Cruz (Fiocruz-Minas), BRAZIL

## Abstract

Although more emphasis has been given to the genetic and environmental factors that determine host vulnerability to malaria, other factors that might have a crucial role in burdening the disease have not been evaluated yet. Therefore, this study was designed to assess the effect of khat chewing on the incidence of severe malaria syndromes and immune responses during malaria infection in an area where the two problems co-exist. Clinical, physical, demographic, hematological, biochemical and immunological data were collected from *Plasmodium falciparum* mono-infected malaria patients (age ≥ 10 years) seeking medication in Halaba Kulito and Jimma Health Centers. In addition, incidences of severe malaria symptoms were assessed. The data were analyzed using SPSS (version 20) software. Prevalence of current khat chewer malaria patients was 57.38% (95%CI =53-61.56%). Malaria symptoms such as hyperpyrexia, prostration and hyperparasitemia were significantly lower (P<0.05) among khat chewer malaria patients. However, relative risk to jaundice and renal failure were significantly higher (P<0.05) in khat chewers than in non-khat chewer malaria patients. Longer duration of khat use was positively associated with incidence of anemia. IgM and IgG antibody titers were significantly higher (P<0.05) among khat chewer malaria patients than among malaria positive non-chewers. Although levels of IgG subclasses in malaria patients did not show significant differences (P>0.05), IgG3 antibody was significantly higher (P<0.001) among khat chewer malaria patients. Moreover, IgM, IgG, IgG1and IgG3 antibodies had significant negative association (P<0.001) with parasite burden and clinical manifestations of severe malaria symptoms, but not with severe anemia and hypoglycemia. Additionally, a significant increment (P<0.05) in CD4^+^ T-lymphocyte population was observed among khat users. Khat might be an important risk factor for incidence of some severe malaria complications. Nevertheless, it can enhance induction of humoral immune response and CD4^+ ^T-lymphocyte population during malaria infection. This calls for further investigation on the effect of khat on parasite or antigen-specifc protective malaria immunity and analysis of cytokines released upon malaria infection among khat chewers.

## Introduction

Malaria remains one of the most widespread diseases affecting human race in tropical and subtropical regions of the world. It is caused by five different species of *Plasmodium* parasites [1] and transmitted by female Anopheles mosquito. *Plasmodium falciparum* and *P*. *vivax* are the main malaria parasites in most malaria endemic areas, with *P*. *falciparum* being more pathogenic. According to the World Health Organization (WHO) report [2], of all malaria cases in the world, 60% were occurring in Africa with 75% of global *P*. *falciparum* malaria cases, from which 80% mortality was documented. In Ethiopia, the major proportion of the total area (75%) is malarious with 68% of the total population living in areas at risk of malaria [3, 4]. Malaria prevalence and transmission in Ethiopia depends on altitude and rainfall [5, 6].

Khat (*Catha edulis*, Forsk), slow-growing shrub or tree believed to be native to Ethiopia, is commonly used for social and recreational purposes [7]. In 1980, the World Health Organization (WHO) classified this plant as drug of abuse that can produce mild to moderate psychological dependence [8]. In the past, it was restricted to only some groups of a population. However, nowadays, the habit of khat chewing is known in Europe, North America, Australia, and Canada; it is becoming an everyday psychoactive drug used by most of the populations in east Africa, Saud Arabia and Yemen [9–11]. In Ethiopia, reports from Butajira and Jimma areas show that there is a tremendous increase of khat use among the publics, students and teachers at school, and students and instructors at universities [12–14].

Khat is naturally grown and chewed in areas at altitude ranging from 1500–2000 meter above sea level (masl) [15]. In Ethiopia, such areas are known as malaria endemic although occasional transmission can occur in areas with altitude >2,000masl [16]. In recent report from community based survey conducted on prevalence of khat use in Halaba Kulito Town where malaria is a major causes of health problem, the highest prevalence of khat chewing (57%) from Ethiopia was documented [17]. This shows that most malaria patients in this area might have frequent exposure to khat. Besides, the plant has been used for treatment of various illnesses such as influenza, stomachache, asthma, gonorrhea, malaria and vomiting among some communities [17, 18]. Some endogenous people of East Africa and the Meru tribe of Kenya also use khat for treatement of malaria [18–21]. Likewise, some people in Yemen have been using khat for treatment of obesity and suppression of appetite besides its role in alleviation of headaches through inhalation of fumes of burning khat leaves [22].

Even though khat has been used either for stimulation purpose or its medicinal value for treatment of malaria, to the authors knowledge, its effect on outcome of malaria infection and crucial immune responses during malaria infection in human is not known. This prompted this study which aimed to assess incidence of severe malaria complications among khat chewer *P*. *falciparum* patients and their immune responses in malaria-stricken areas.

## Materials and Methods

### Study sites and period

**Fig 1:**
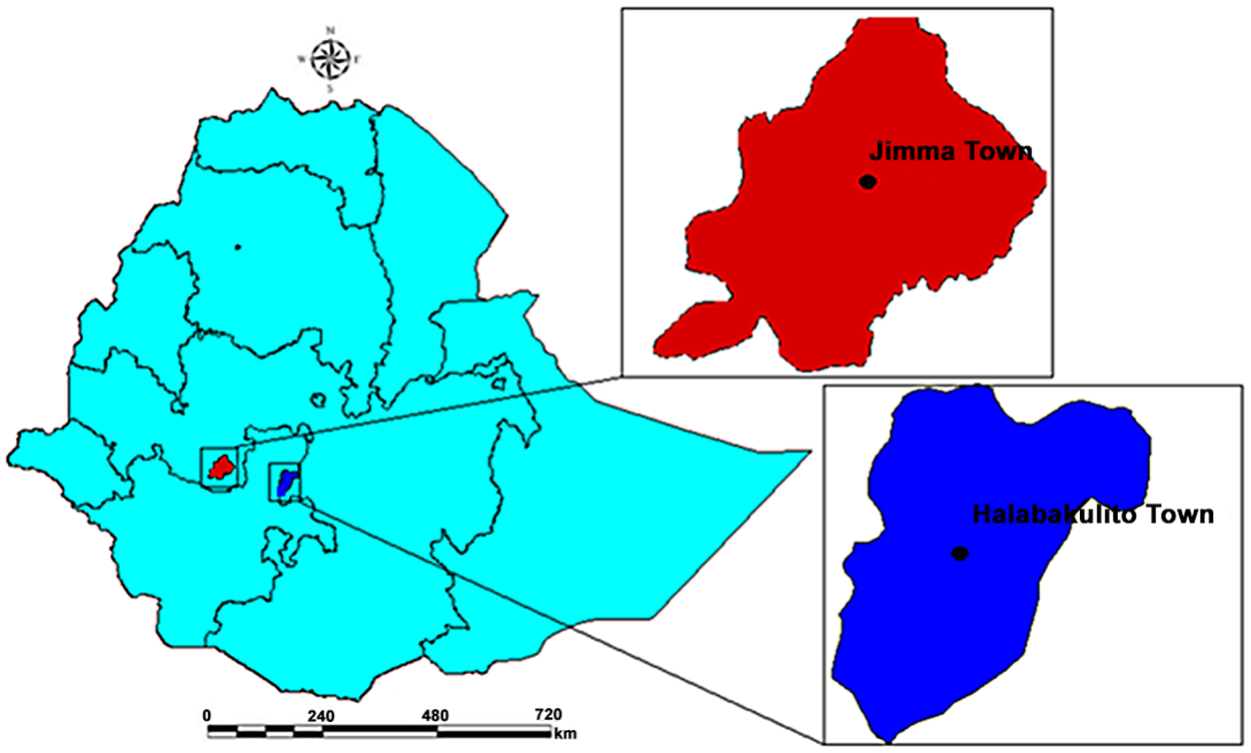
Map of the study sites: Halaba Kulito Town (South Ethiopia) and Jimma Town (Southwest Ethiopia).

The study was conducted at Jimma and Halaba Kulito Health Centers from July 2012 to December 2013 (Fig 1). The study sites, Halaba Kulito (Southern Ethiopia) and Jimma Town (Southwest Ethiopia) are geographically located at altitudes ranging from 1554–2149 and 1780 masl, longitude of 38° 7' 0" E and 36°50’E, and 7° 18' 0" and 7°41’N latitudes, respectivly. Furthermore, the annual rainfall and temperature of Halaba Kulito and Jimma Town range between 857–1085 and 1138–1690mm, and 17–20 and 14–30°C, respectively [23]. Even though the overall malaria prevalence is showing a sort of declining trend nationwide [24], malaria is still the major health problem in the districts, and *Anopheles arabiensis* is the main vector [25]. The study areas were purposely selected due to the high prevalence of khat chewing practice and malaria endemicity.

### Study population and sample size

Presumptive malaria patients seeking medication in the health centers were examined by medical laboratory technicians for malaria infection following standard parasitological procedures. The inclusion criteria used for enrollment were: malaria patients aged ≥10 years [this age was taken as cut off point in this study as, culturally, children more than 10 years are allowed to chew khat with their parents in this specific community (personal communication)], and mono-infected with *P*. *falciparum*. Accordingly, a total of 366 malaria patients (n = 266 from Halaba Kulito and n = 100 from Jimma Health Center) were recruited in the study on availability basis. Medication with any anti-malarial drugs (for the current illness) prior to the study, pregnancy, and admission to anti-retroviral therapy (ART) or tuberculosis (Tb) clinics were considered as an exclusion criteria.

### Data collection procedure

Prior information on socio-demographic characteristics, and frequency and duration of khat use among patients were assessed using mini-structured interview questions. Moreover, clinical data on malaria syndromes (complicated and uncomplicated) were assessed by health professionals working in the selected health centers. Nutritional status of each patient was checked by measuring his/her body mass index (BMI) using their height and weight values. Classification of patients based on BMI was made following WHO classification criteria [26]. Accordingly, those with BMI <16 were classified under severe thinness, 16–16.99 as moderate thinness, 17–18.49 as mild thinness, between 18.5 and 24.99 as normal range and ≥25 as overweight.

### Laboratory analysis

After written consents were obtained from the study participants or guardians of the patients with complicated severe malaria and children <18 years, blood samples were collected. Thin and thick blood smears were prepared in duplicate per patient for microscopic examination. Having fixed only the thin smear in methanol, the whole smears were stained with 10% Giemsa (pH = 7.2) for 10 min. Based on the morphological appearance of the parasite in infected red blood cells (iRBCs), parasite identification was carried out under a microscope (oil immersion objective, 100X). Parasite load was calculated by counting the number of asexual stage parasites per 200 white blood cells (WBCs), assuming mean human WBC is 8,000/μL [27]. Each blood smear was examined by experienced laboratory technician in the health centers and then re-checked by a certified laboratory technician at Jimma University. The degree of parasitemia was graded in detail as mild, moderate and severe, when a count was between 1–999 parasite/μL, 1000–9999/μL, >10000/μL, respectively [28]. It was considered as hyperparasitemia when the parasite load was >100,000 parasite/μL [29].

About 5mL of venous blood samples were collected from each study participant by vein puncture of the antecubital vein using a 21 gauge hypodermic sterile needle and syringe for different analyses. The blood samples were then transferred into clean sterile EDTA pre-coated centrifuge tubes. Few drops of blood samples (~10μL) were taken for measurement of blood glucose level using Hemocue instrument (Hemocue Glucose 201 analyzer, Angelholm, Sweden). After completing the sampling and data collection, participants with non-complicated severe malaria, hence not admitted to the health centers, were treated with artemether-lumefantrine (20mg base/kg for three days twice a day); but admitted patients were treated with IM quinine (10mg base/kg every 8 hours for five days) as per the recommendations of the National Malaria Treatment Guideline [30].

#### Hematological and biochemical tests

From the collected blood samples, small quantity (~10μL) were used for quantification of total WBCs, lymphocytes, RBCs, hematocrit (HCT), hemoglobin (Hb) and platelets using CBC machine [Automated complete blood cells (CBC) Analyzer: Sysmex KX-21]. Some of the venous blood samples (~1mL) were centrifuged at 10,000 rpm for 10 minutes at room temperature; the sera were transferred into new eppendorf tubes and stored at -80°C until processed. Then, the sera were used for determination of liver biomarkers: serum glutamic oxaloacetate transaminase (sGOT), serum glutamic pyruvic transaminase (sGPT), total bilirubine and albumin levels. In addition, kidney function tests such as measurement of creatinine and urea and inflammation biomarkers such as uric acid (UA) and C-reactive protein (CRP) were quantified using automated immunochemical analyzer (Axsym MEIA 3^rd^ Generation).

### Classification of severe malaria syndromes

Severe malaria symptoms were classified following WHO guidline for the management of severe malaria [29] as impaired consciousness, comma, prostration (inability to sit unassisted), multiple convulsions, cerebral malaria (when coma persists for at least 30 minutes after a generalized convulsion), abnormal bleeding, jaundice (serum bilirubin concentration of >3mg/dL), severe anemia (haemoglobin level <5 g/dL in children and <7g/dL in adults), renal failure (serum creatinine level >3mg/dL), hypoglycaemia (serum glucose level <40 mg/dL), hyperparasitaemia, hyperpyrexia (body temperature ≥40°C), respiratory distress and circulatory collapse [29].

### Immunological assay

Blood samples collected from a total of 360 study participants were used for this assay. Briefly, the study participants were divided into four categories; (i) parasitologically confirmed *P*. *falciparum* positive with clinical manifestations of malaria infection and aged ≥10 years but non-khat chewers (n = 120), (ii) parasitologically confirmed *P*. *falciparum* positive with clinical manifestation of malaria infection, self-reported khat chewers and aged ≥10 years (n = 120), (iii) neither malaria infected nor khat chewers and aged ≥10 years (n = 60), (iv) khat chewer and aged ≥10 years, negative for malaria infection under microscope and rapid diagnostic test (RDT) (n = 60). About 2mL of the venous blood samples were centrifuged at 10,000 rpm for 10 minutes at room temperature; the sera were transferred into new eppendorf tubes and stored at -80°C until processed. Finally, the sera were used for quantification of serum immunoglobulins (Ig): IgG, IgM, IgG1, IgG2, IgG3, and IgG4 using nephelometric assay [31]. Serum Ig class and the IgG subclass levels were expressed in g/L.

The remaining blood sample (~1mL) was used for quantification of T-lymphocytes population: CD4^+^ (T-helper cells), CD8^+^ (cytotoxic T-cells) using flow cytometric technology. Accordingly, 100μL of Fluoroisothiocyanate (FITC)-conjugated anti-CD4 and anti-CD8 monoclonal antibodies produced in mouse (Sigma Aldrich) that react against receptors of CD4^+^, and CD8^+^ were added directly to 100μL of whole blood, which were then lysed using fluorescence-activated cell sorter (FACS) lysing solution (1 in 10 ddH_2_O). Following centrifugation at 4°C for 5 minutes at 1500 rpm, samples were re-suspended in PBS (pH = 7.4) and analyzed directly using flow cytometer [32].

In this study, potential environmental confounding factors including exposure to other suppressive substances such as alcohol and cigarette smoking, and presence of chronic illness due to Tb and HIV were carfully assessed by health professionals by checking for their admission to Tb or ART clinic in the health centers or elsewhere. Whenever there was a suspect, the collected blood samples were checked on spot for sero-positivity to HIV infection (using HIV 1/2 Stat-Pak rapid diagnostic, USA). In addition, urine pregnancy test (using HCG test kit, ADVOCARE Pharma, China) was conducted for all female participants of reproductive ages. With regard to smoking and habit of drinking alcohol, all the data were based on participants self report. As the researchers attested during enrollment, most of the khat chewer malaria patients were observed engaged in khat chewing even in the premises of the health centers.

### Data analysis

Data were checked for their completeness, correctness, and then carefully entered into Microsoft Office Excel (2007) sheet. Frequency, percentage and means of different variables were analyzed using descriptive statistics. Furthermore, the data were analyzed using SPSS software version 20. Univariate and multivariate logistic regression models were used to show the effect of independent variables (khat chewing, duration and frequency of chewing) on incidence of dependent variables (severe malaria symptoms). Pearson and Spearman’s rank correlations were employed to analyze association between severe malaria symptoms and immune responses, and interaction between antibodies respectively. Hematological, biochemical and immunological parameters between khat chewer and non-chewer malaria patients were compared using Chi-square test. Independent variables were compared by Mann-Whitney U test. Significance level was considered at least at 95% confidence interval in all data analysis.

### Ethical consideration

The study was ethically approved by the Research and Ethical Review Committee of Jimma University, College of Health Sciences. Written consent/assent was obtained from each participant or their guardians (for children less than 18 years and complicated severe malaria) prior to data collection. In addition to these, all the study participants were examined and treated in line with the national malaria guidelines of the Ethiopian Federal Ministry of Health.

## Results

**Fig 2:**
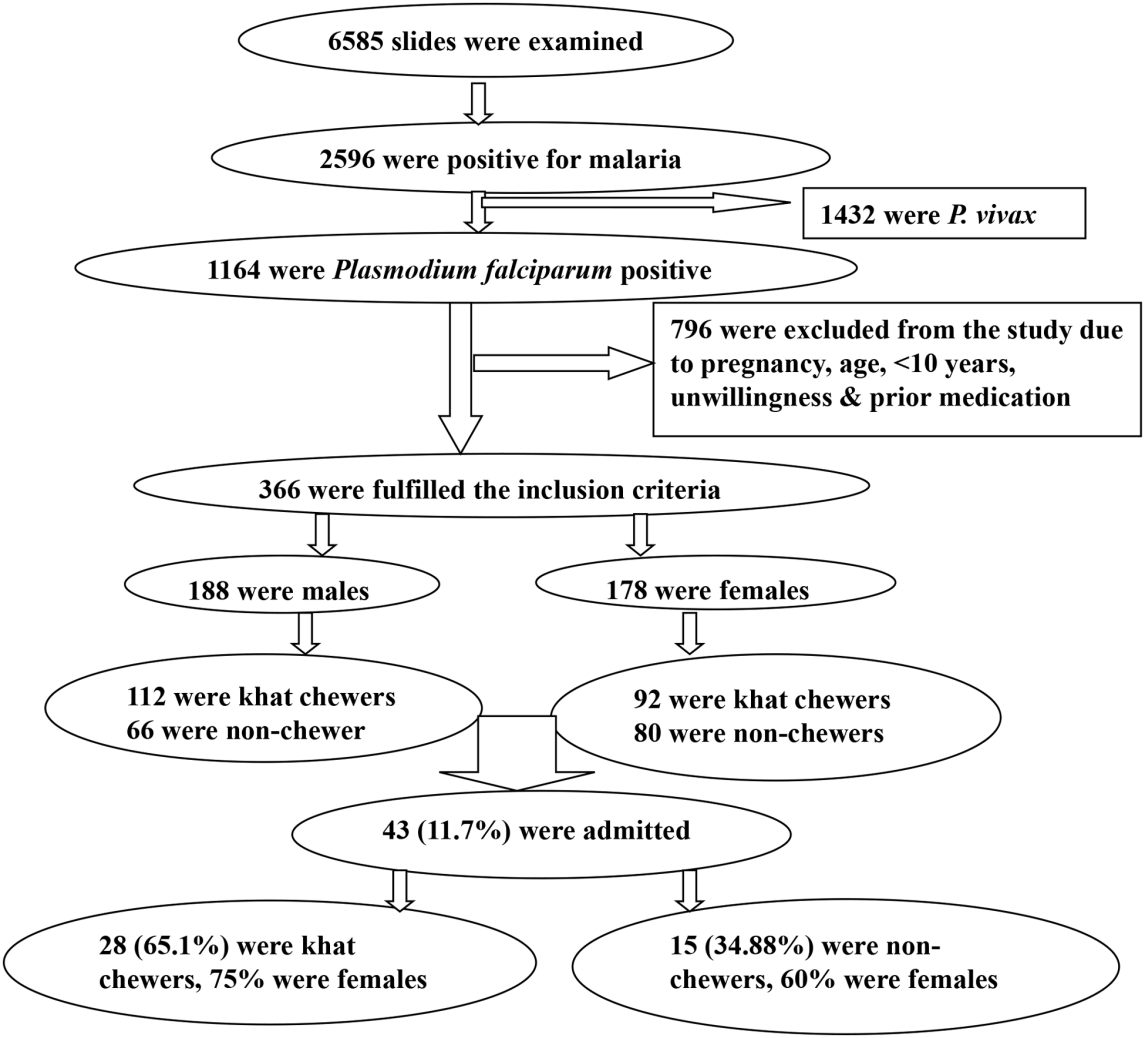
Flow chart used in recruitment of the study participants, Halaba Kulito District (South Ethiopia) and Jimma Town, (Southwest Ethiopia).

During the study period, a total of 6585 presumptive cases were examined for malaria infection. About 2596 (39.42%) of them were microscopy-positive for malaria infection, of which 1432 (55.16%) were infected with *P*. *vivax* and the rest had *P*. *falciparum* infection. About 798 (68.5%) *P*. *falciparum* positive patients were excluded from the study for reasons such as age (children <10 years), pregnancy and prior medication. Thus, only 366: 188 (51.36%) males and 178 (46.63%) females who fulfilled the inclusion criteria were enrolled in the study (Fig 2). Among 366 screened malaria patients, 198 (54.1%) were khat chewers, 156 (42.62%) were non-khat chewers and 12 were concurrent users of khat and tobacco. The median age of the participants was 26.5 years.

### Khat chewing and malaria status

Among the 366 *P*. *falciparum* malaria positive patients enrolled in the study, 210 (57.38%, 95% CI = 53–61.56%) were self-reported khat chewers. About 12 (5.71%) of these were concurrent users of khat with tobacco. Significant difference was not observed between sexes for the habit of khat chewing (OR = 1.32, 95% CI = 0.76–2.31). That is the number of males and females malaria patients involved in khat chewing were comparable. The majority (55.23%) of khat chewers were in the age range of 20 to 30 years. With regard to frequency of use and starting age, a total of 132 (62.8%) and 105 (50%) of the khat chewer malaria cases were daily users and started khat chewing in at the ages of between 15 and 20 respectively. Analysis on frequency of khat use revealed that malaria patients with daily khat chewing habit were significantly higher than less frequent users (OR = 4.5, 95% CI = 2.49–8.15). Average duration of use of khat was 10.23 ±7.1 years, among which about 99 (47.14%) had exposure years from 15 to 20 (Table 1).

**Table 1:** Khat chewing pattern of malaria patients enrolled in the study in some malaria endemic areas of Ethiopia.

Characteristics of chewers	Alternatives	Proportion (%)
Sex	Male	112 (53.33)
	Female	98 (46.67
Age category (year)	<20	30 (14.28)
	20–30	116 (55.23)
	31–40	38 (18.09)
	>40	26 (12.38)
Frequency of khat use	Daily	132 (62.8)
	Every other day	44 (21)
	Once/week	34 (16.2)
Khat chewing start age	<15 years	34 (16.2)
	Between 15 and 20	105 (50)
	Between 20 and 30	38 (18.1)
	>30	33 (15.7)
Khat chewing duration (year)	0–5	54 (25.7)
	6–10	37 (17.62)
	11–12	99 (47.14)
	>20	20 (9.5)

### Clinical characteristics of the study participants

Even though all participants had a history of fever at least for two days prior to diagnosis, only 284 (77.59%) of them had an axillary temperature ≥37.5°C during recruitment with an average of 4.15 days of illness. Geometric mean parasite count (asexual stage) was 6932.3 parasite/μL. Average body temperature recorded was 38.01°C. In this study, a total of 221 (60.38%) participants responded that they had bed nets, but they might not use them properly. According to the respondents’ statements, they use bed net only when the occurrence of mosquitoes increases in their locality (Table 2).

**Table 2:** Characteristics of the study participants in some malaria endemic areas of Ethiopia.

Characteristics	Proportion
Age (median)	26.5 year
Febrile cases during enrollment	284 (77.59%)
Average days of illness	4.15 (2–7days)
Vomiting	197 (53.8%)
Diarrhea	89 (24.31%)
Mean duration of exposure to khat	11.23 ±7.1 (0.25–30 years)
Bed net coverage	221 (60.38%)

The most commonly observed symptoms were high-grade fever with rigors/chills, headache, sweat, loss of appetite, fatigue or weakness, nausea, vomiting, and cough. The proportion of patients with severe parasitemia (>10,000 parasite/μL) was 175 (47.81%). Mean BMI of the participants was 19.43 ±2.4, from which 121 (33.06%) of them were underweight, 40 (10.92%) were in severe thinness range, BMI <16. Very few patients, 16 (4.37%) were overweight and found in pre-obesity category. Participants whose BMI value from 16 to 16.99 were significantly higher (P = 0.019) amon non-chewer malaria patients (Table 3).

**Table 3:** Proportion (%) of BMI and parasite load of khat chewer and non-chewer malaria patients in some malaria endemic areas of Ethiopia.

Clinical features	Parameters	Patient status		
		Khat chewers	Non-chewers	P. value
BMI (kg/m^2^)	<16	25 (11.9)	15 (9.6)	0.48
	16–16.99	6 (2.85)	13 (8.33)	0.019
	17–18.49	32 (15.24)	30 (19.23)	0.31
	18.5–24.99	138 (65.7)	90 (57.7)	0.11
	≥25	9 (4.3)	7 (4.5)	0.92
Parasite load/μL	1–999	33 (15.7)	25 (16)	0.93
	1000–9999	80 (38.1)	53 (50)	0.41
	>10000	97 (46.2)	78 (21.3)	0.47

### Hematological and biochemical characteristics

Mean Hb and blood glucose levels measured among the study participants were 14.1g/dL and 117.4 mg/dL respectively. Mean values of other hematological parameters such as WBCs, RBCs, HCT, platelets and lymphocytes were 5.41*10^3^/μL, 4.35*10^6^/μL, 37.31%, 253*10^3^/μL and 49.3% respectively. Level of mean liver function biomarkers such as liver enzymes, serum GPT and serum GOT, and others; albumin, and total bilirubin were 30.67 IU/L, 33.45 IU/L, 3.38g/dL, and 1.29mg/dL respectively. Average serum urea and creatinine level were 41.8g/dL and 1.29 mg/dL respectively (Table 4). Also, liver and kidney function tests between khat chewer and non-khat chewer malaria patients revealed that the level of liver enzymes (serum GOT and serum GPT) was significantly higher (P<0.001) among khat chewer than among non-khat chewer malaria patients. Also significant reduction of albumin level (P = 0.027) was observed among khat chewer malaria patients. Level of serum creatinine was significantly higher (P = 0.002) among chewer malaria patients. Bilirubin level observed in most khat users malaria patients (65.8%) was above normal range (>1.2mg/dL) but only in five khat chewer malaria patients serum bilirubin level >3mg/dL (Jaundice) was measured. In addition, one of the two inflammation biomarkers measured (CRP) showed significantly higher level (P<0.001) among khat chewer malaria patients. RBC, WBC, and HCT levels and BMI were not significantly different (P>0.05) between the two groups. Among hematological parameters evaluated, only platelet count indicated significant decrement (P<0.001) among khat chewer malaria patients (Table 4).

**Table 4:** Comparison of physical, clinical, biochemical and hematological features (Mean ±SD) between khat chewer and non-chewer malaria patients, in some malaria endemic areas of Ethiopia.

Parameters	Level ±SD (range)	Chewers (mean ±SD)	Non-chewers (mean ±SD)	P-value
Hemoglobin level (g/dL)	14.1±2.04 (9.2–17.9)	14.31±2.12	14.00±1.94	0.562
Blood glucose level (mg/dL)	117.4±21.37 (62–178)	112.21±29.8	119.05±18.9	0.244
BMI (kg/m2)	19.73±2.4 (13.6–27.1)	20±2.42	19.22±2.755	0.75
WBC *103/μL	5.41±1.13 (4.8–6.8)	5.09±1.23	5.6±0.98	0.303
RBC *106/ μL	4.35±1.08 (4.41–5.7)	4.26±0.66	4.67±0.73	0.377
HCT (%)	37.31±8.31(32.3–46.1)	33.2±5.43	40.8±9.17	0.922
Platelet *103/ μL	253±87.2 (107–387)	211.6±16.14	281.2±25.3	0.000*
Lymphocytes (%)	49.3±5.455 (33–58)	48.51±11.84	50.3±16.1	0.532
Temperature (°C)	37.58 ±1.1 (35.6–41.2)	37.2 ±1.17	37.96±1.14	0.031*
Parasite count (parasite/μL)	7321.2± 57810 (320–280,000)	5920 ±32513	7768 ±43718	0.000*
Albumin (g/dL)	3.38 ±0.77 (2.14–4.81)	3.16±0.5	3.59±0.58	0.027*
Creatinine (mg/dL)	1.29±0.17 (0.5–2.31)	1.43±0.22	0.98±0.19	0.002*
Urea (mg/dL)	41.8±9.28 (22–87.9)	42.44±9.56	40.5±8.3	0.0326*
SGOT (IU/L)	33.45±13.3 (15.16–86)	36.01±10.4	30.4±15.7	0.000*
SGPT (IU/L)	30.67±10.79 (15.03–72)	34.09±11	27.95± 8.75	0.000*
Total bilirubin (mg/dL)	1.29±0.32 (0.11–2.01)	1.28±0.22	1.22±0.42	0.042*
CRP (mg/dL)	4.4±3.9 (0.99–12.4)	7.35±1.05	1.36±0.63	0.000*
Uric acid (mg/dL)	4.84±0.76 (4.2–5.6)	5.12±0.84	4.6±0.74	0.26

* significant difference between patients of khat chewers and non-chewers. Total of the sample analyzed for platelet, WBC, RBC, HCT and lymphocyte counts were 174 (n = 91 were khat chewers; n = 83 were non- chewer malaria patients).

### Incidence of severe malaria syndromes among khat chewers

Relative risk of khat chewer malaria patients to severe malaria complications was compared against non-khat chewers. Accordingly, risk of hyperparasitemia, prostration, impaired consciousness and hyperpyrexia were significantly higher (P<0.05) among non-khat chewer malaria patients. Severe anemia, and hypoglycemia were not observed in any of the study participants. Except in few khat chewer malaria patients (6.8%) with jaundice condition, significant differences was not observed (P>0.05) between the two groups (Table 5).

**Table 5:** Comparative incidence of severe malaria symptoms among khat chewer and non-chewer malaria patients in some malaria endemic areas of Ethiopia.

Characteristics	Patients status		
	Non-khat chewer (%)	Khat-chewer (%)	P. value
Hyperpyrexia (≥ 40°C)	16 (10.26)	0 (0)	0.000*
Prostration	65 (41.67)	29 (13.81)	0.018*
Hyperparasitemia (>100,000 parasite/μL)	25 (16)	12 (5.71)	0.001*
Impaired consciousness	15 (9.6)	28 (13.3)	0.047*
Mild jaundice (Total bilirubin >2.5mg/dL)	11 (7.05)	21 (10)	0.296
Respiratory distress	17(10.89)	33(15.7)	0.379
Hypoglycemia (glucose level <40mg/dL)	0 (0)	0 (0)	
Abnormal bleeding	0 (0)	0 (0)	
Renal problem (creatinine >3mg/dL)	0 (0)	0 (0)	
Multiple convulsions	0 (0)	0 (0)	
Severe anemia (Hb level <5 or 7g/dL)	0 (0)	0 (0)	

* significant difference between patients of khat chewers and non-chewers malaria patients.

During the study period, some patients with severe malaria complications, 43 (11.7%), were admitted to the health centers. Among these, 15 (34.88%) were non-chewer and 28 (65.1%) were khat chewer malaria patients, where the number of admitted khat chewers were significantly different (OR = 3.45, 95%CI, 1.93–6.16) from the non-chewer malaria patients. Moreover, the proportion of admitted female malaria patients was higher than that of males in both groups, 9(60%) non-khat chewers and 21 (75%) chewers, with the proportion being significantly higher (OR = 4.32, 95%CI, 2.43–7.68) among the khat chewers. There was no death report from the two categories (Fig 2).

### Severe malaria symptoms independently associated with khat chewing

Based on logistic regression tests, variables that are associated with khat chewing were age, body temperature, prostration, parasite load, total bilirubin and creatinine. As age increases, the habit of khat use was significantly higher (OR = 1.034, 95%CI = 1.00–1.068). Khat chewing per se might not be a risk factor for incidence of hypoglycemia and severe anemia in malaria patients as no significant change was observed in glucose and Hb levels between chewer and non-chewer malaria patients (P>0.05). But longer duration of khat use significantly increased anemic condition in malaria patients (OR = 2.2, 95% CI = 1.07–4.5) (Tables 6 and 7). On the other hand, khat chewing has a negative association with some severe malaria symptoms such as high fever (hyperpyrexia), prostration and hyperparasitemia. These syndromes were exceedingly evidenced among non-khat chewer malaria patients. Even as khat use increases (longer duration), significant reduction of febrile cases (OR = 0.409, 95% CI = 0.167–1.00), parasite load (OR = 0.108, 95% CI = 0.021–0.547), and prostration (OR = 0.128, 95% CI = 0.036–0.462) were observed in malaria patients. However, frequent khat chewing per se in malaria patients could be a risk factor to jaundice and renal impairment as elevated level of liver and kidney biomarkers such as total bilirubin, (OR = 3.68, 95% CI = 1.699–7.977) and creatinine (OR = 1.34, 95%CI = 0.5–3.565) respectively was observed (Tables 6 and 7).

**Table 6:** Univariate and multivariate logistic regression model assessing variables related to khat chewing in *P*. *falciparum* infected malaria patients in some malaria endemic areas of Ethiopia.

Variables	Parameters	Total	No (%) of khat chewer malaria cases	COR (95% C.I)	P-value	AOR (95% C.I)	P-value
Age	NA	366	210 (57.38)	1.07 (1.038–1.103)	0.000	1.034 (1.00–1.068)	0.044
Temp (°C)	< afebrile	188	169 (89.9)	1.00		1.00	
	>febrile	178	41 (23.03)	0.135 (0.079–0.23)	0.000	0.102 (0.042–0.249)	0.000
Prostration	No	272	181 (66.54)	1.00		1.00	
	Yes	94	29 (30.85)	0.46 (0.286–0.74)	0.001	0.362 (0.151–0.867)	0.023
Parasite load	<2.5%	329	198 (60.18)	1.00		1.00	
	>2.5%	37	12 (32.43)	0.105 (0.043–0.26)	0.000	0.108 (0.021–0.547)	0.007
Albumin	3.4–4.8	196	92 (46.94)	1.00		1.00	
	<3.4	170	108 (63.53)	2.665 (1.655–4.29)	0.000	2.154 (0.805–5.76)	0.012
SGOT(IU/L)	0–45	237	124 (52.32)	1.00		1.00	
	>45	129	76 (58.9)	18.7 (7.91–44.13)	0.000	11.75 (2.59–53.23)	0.001
SGPT(IU/L)	0–50	240	113 (47.1)	1.00		1.00	
	>50	126	87 (69.04)	5.526 (3.244–9.41)	0.000	5.439 (3.11–8.77)	0.000
TB (mg/dL)	0.1–1.2	189	75 (39.68)	1.00		1.00	
	>1.2	177	125 (70.62)	6.63 (4.115–10.69)	0.000	5.757 (2.56–12.94)	0.000
Urea(mg/dL)	18–55	224	97 (43.3)	1.00		1.00	
	>55	144	103 (71.53)	10.169 (5.74–18.016)	0.000	14.5 (4.85–43.36)	0.000
Creatinine(mg/dL)	0.6–1.2	138	53 (38.4)	1.00		1.00	
	>1.2	228	147 (64.47)	4.528 (2.88–7.117)	0.000	1.34 (0.5–3.565)	0.027
Platelet count (cells/μL)	>150,000	140	66 (47.14)	1.00		1.00	
	<150,000	34	25 (73.53)	9.337 (5.99–14.54)	0.000	8.7 (5.476–13.83)	0.000

NB: COR = crude odd ratio, AOR = adjusted odd ratio, there was no multicolinarity in the model and on case-wise residue diagnosis, value greater than three standard deviation was not encountered. NA = not applicable. The catagories of the predictive variables that received odd ratios of 1.00 are reference catagories

**Table 7:** Variables associated to longer duration of khat use in *P*. *falciparum* infected malaria patients in some malaria endemic areas of Ethiopia.

Characteristics		Total khat users	No (%) with longer duration of khat use	COR (95% C.I)	P. value	AOR (95% C.I)	P-value
Hb (g/dL)	Non-anemic	156	74 (47.4)	1.00		1.00	
	Anemic	54	36 (66.67)	2.28 (1.29–4.06)	0.031	1.89 (1.27–5.24)	0.002
Temp (°C)	< afebrile	169	98 (58)	1.00		1.00	
	>febrile	41	12 (29.27)	0.308 (0.18–0.536)	0.000	0.41 (0.17–1.002)	0.05
Prostration	No	181	103 (56.9)	1.00		1.00	
	Yes	29	7 (24.14)	0.262 (0.125–0.536)	0.000	0.128 (0.036–0.46)	0.002
Vomiting	No	135	73 (54)	1.00		1.00	
	Yes	75	37 (49.33)	0.808 (0.477–1.37)	0.001	0.124 (0.038–0.408)	0.027
Platelet count (cells/μL)	>150,000	66	34 (51.5)	1.00		1.00	
	<150,000	25	18 (72)	2.47 (1.371–4.16)	0.000	1.779 (1.29–2.45)	0.001
WBC/ μL	>4*10^3^	54	33 (61.1)	1.00		1.00	
	<4*10^3^	37	29 (78.38)	2.27 (1.22–4.217)	0.009	1.554 (0.763–3.164)	0.027

NB: COR = crude odd ratio, AOR = adjusted odd ratio, there was no multicolinarity in the model and on case-wise residue diagnosis, value greater than three standard deviation was not encountered. The catagories of the predictive variables that received odd ratios of 1.00 are reference catagories

Furthermore, other variables such as platelet count, levels of albumin, GOT, GPT, urea, WBC and Hb, but sex were found as strongly associated with khat chewing. Accordingly, frequent khat chewing habit in malaria patients was strongly associated with significant elevated level of GOT (OR = 11.75, 95%CI = 2.59–53.23), GPT (OR = 5.439, 95%CI = 3.11–8.77), albumin (OR = 2.154, 95% CI = 0.805–5.76), platelet count (OR = 8.7, 95%CI = 5.476–13.83) and urea (OR = 14.5, 95%CI = 4.85–43.36) (Table 6). Besides severe malaria complications, frequent khat chewing per se in malaria patients could be a risk factor for elevated level of liver and kidney biomarkers such as GOT, GPT, albumin, and urea, and platelet count (Table 6)

### Effect of khat on humeral immune responses

**Fig 3:**
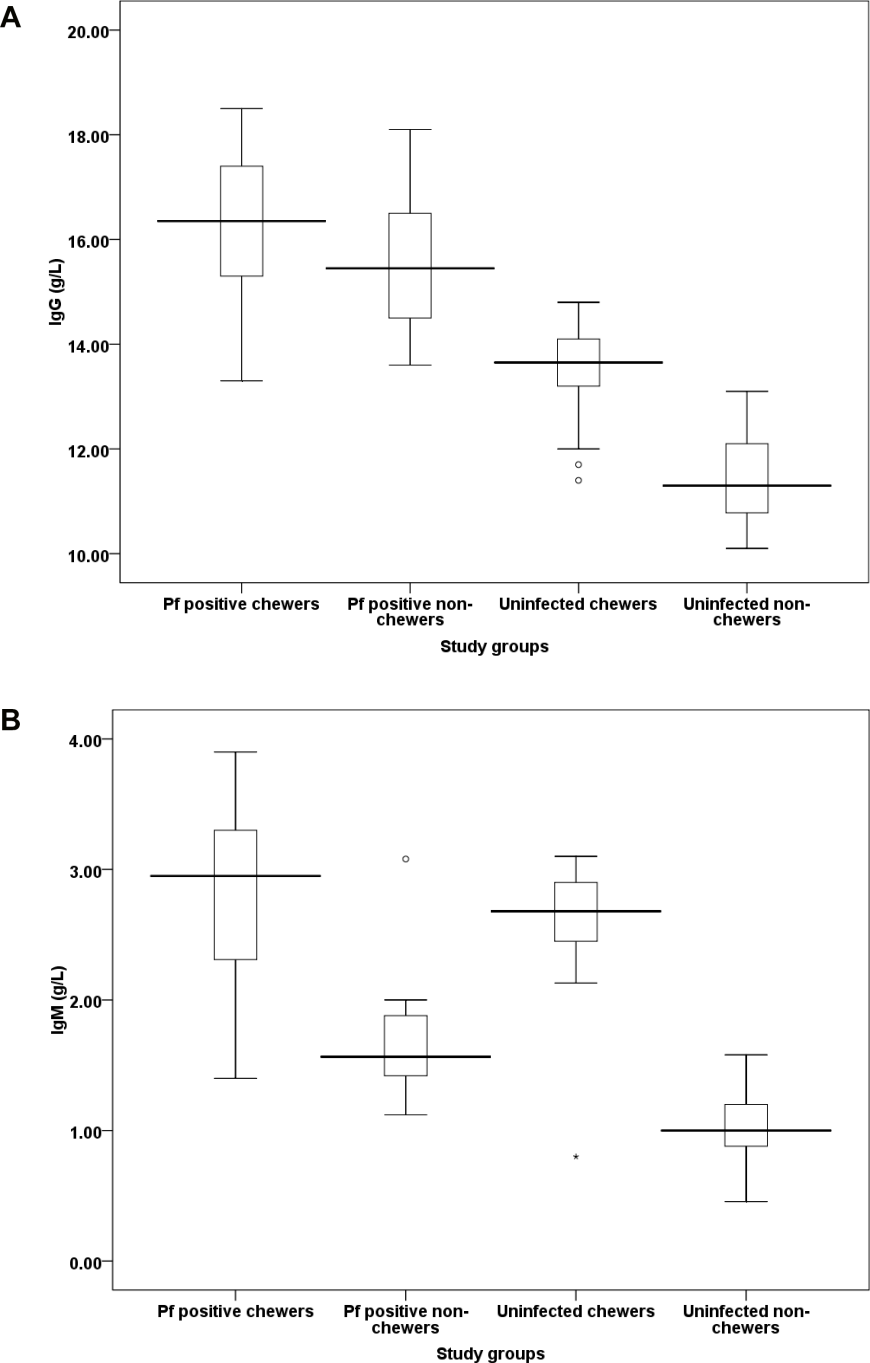
Levels (mean ±SD) of antibodies’ IgG (a) and IgM (b) response among the study participants in some malaria endemic areas of Ethiopia. Values with asterisk are significantly different (Mann-Whitney U test) from values of *P. falciparum* positive non-chewers or neither infected nor chewer.

The result of this study revealed positive association between khat chewing and malaria infection against the level of antibody titer with regard to IgG. The association was more pronounced among infected khat chewer individuals. Even in the non-infected individuals, the antibody titer was higher among khat chewers showing the significance of effect (P<0.001) of exposures to the plant on the level of antibody titer of an individual (Fig 3a). On the other hand, the level of IgM titer appears to depend more on whether the individual is chewer or not than on being infected or not. Accordingly, more IgM titer was recorded from uninfected chewers than from malaria positive non-chewer. Like the case of IgG, however, combination of infection and chewing resulted in significantly higher (P<0.001) IgM titer (Fig 3b)

**Fig 4:**
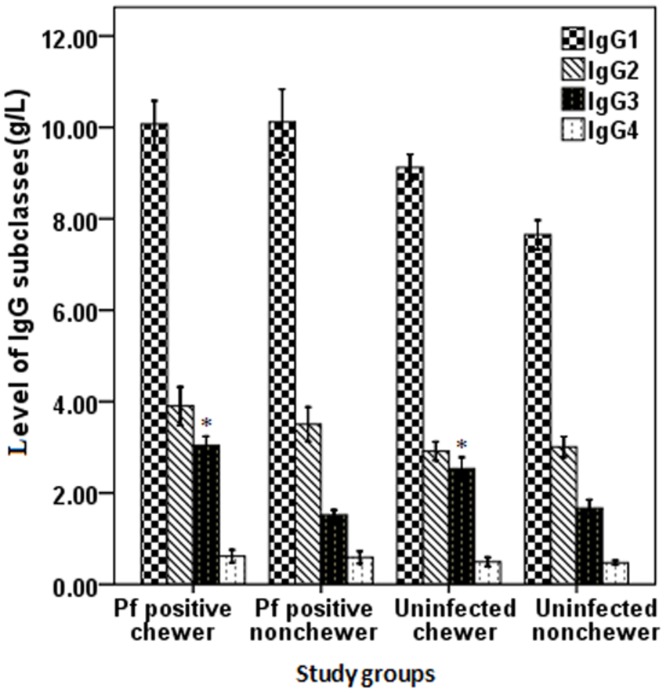
Levels (Mean ±SD) of IgG subclasses; IgG1, IgG2, IgG3 and IgG4 (mean ±SD) among the study participants in some malaria endemic areas of Ethiopia. Values with asterisk are significantly different (Mann-Whitney U test) from values of *P. falciparum* positive non-chewers or neither infected nor chewer.

The levels of IgG subclasses showed different patterns among the study participants. The concentrations of IgG1, IgG2 and IgG4 were not significantly differed (P>0.05) between *P*. *falciparum* positive patients irrespective of being chewer or not. However, the level of IgG3 was significantly higher (P<0.001) among *P*. *falciparum* malaria positive khat chewers than non-chewer malaria patients. IgG3 tends to remain significantly low (P<0.001) in non-chewer cases, infected or not, as compared to chewers. Overall, IgG2 and IgG4 did not show significant difference (P>0.05) among the study participants under all combinations of conditions (Fig 4).

### Association between antibodies and malaria symptoms

According to the Pearson correlation test among khat chewer malaria patients, parasitemia was negatively associated with IgG (r = -0.19, P = 0.037), IgG1 (r = −0.25, P = 0.005) and IgG3 (r = −0.30, P<0.001). As the level of these antibodies (IgG, IgG1 and IgG3) increase, parasite burden among non-khat chewer malaria patients decrease, but, not to IgM, IgG2, and IgG4. Likewise, the high antibodies titer observed among khat chewer malaria patients were also negatively associated with the count of parasitemia. As the level of antibodies such as IgG (r = −0.216, P = 0.018), IgM (r = -0.2, P = 0.028), IgG1(r = -0.186, P = 0.042) and IgG3 (r = -0.23, P = 0.011) increases, significant reduction of parasite count was observed.

Moreover, the elevated level of antibodies titer; IgG, IgG1 and IgG3 among *P*. *falciparum* positive (khat chewer and non-chewer) malaria patients were negatively associated (P<0.05) with severe syndromes of malaria including impaired consciousness, hyperpyrexia, hyperparasitemia and prostration. However, association between the antibodies and severe anemia and hypoglycemia was not observed.

The link interconnecting each of the antibodies assessed in malaria positive (khat chewer and non-khat chewer) patients were investigated using the Spearman’s correlation rank test. As shown in below, multiple positive correlations were identified between antibodies: (IgG *vs* IgM, Spearmen correlation coefficient (r_s_) = 0.529, P<0.01), IgG *vs* IgG subclasses (IgG1, IgG2, and IgG3, r_s_ = 0.587, r_s_ = 0.34, and r_s_ = 0.24 respectively, P<0.01); IgM *vs* IgG subclasses [(IgG2, r_s_ = 0.229, P<0.05), IgG1, and IgG3, r_s_ = 0.427, and r_s_ = 0.698 respectively, P<0.01)] and between IgG subclasses [(IgG1 *vs*.IgG3 (r_s_ = 0.394, P<0.01); IgG2 *vs*. IgG3 (r_s_ = 0.213, P<0.05)]. Although IgG4 was negatively associated with most Igs, significant correlation was not observed (P>0.05).

### Effect of khat on T-lymphocytes

**Fig 5:**
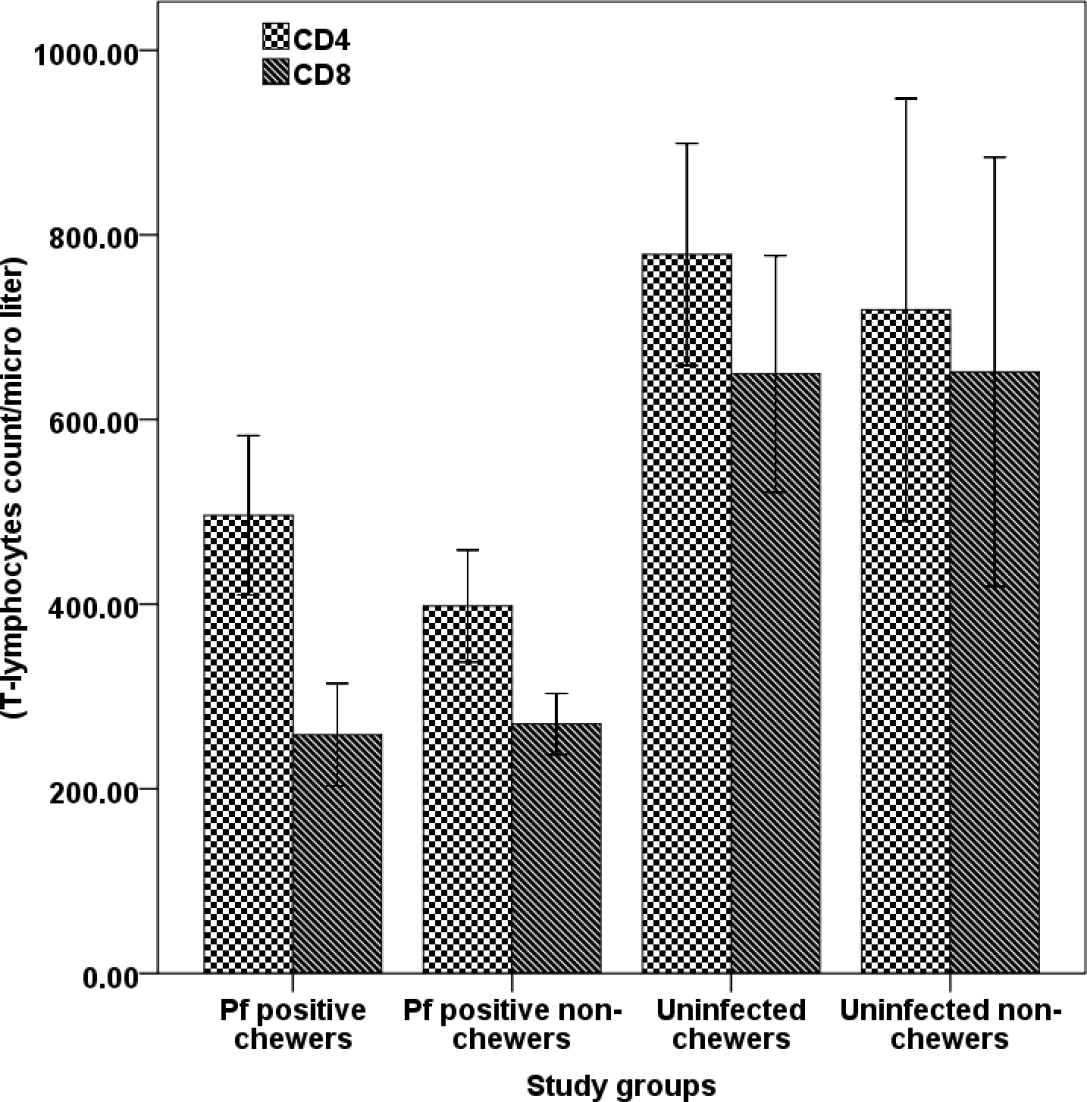
T-lymphocyte population (CD4^+^ and CD8^+^) counts (mean ±SD) of *P. falciparum* infected (self-reported chewers and non-chewer) and healthy (self-reported chewer and non-chewer) participants. Value with asterisk is significantly different (Mann-Whitney U test) from values of *P. falciparum* positive non-chewers.

T-lymphocytes count assessment carried out among different groups of the study participants revealed that, except helper T-lymphocytes or CD4^+^ count among khat chewer malaria patients’, khat did not cause any effect on others. Hence, CD4^+^ count observed among khat chewer malaria patients was significantly higher (P<0.05) from non-chewer malaria positive patients. Likewise, although there was slightly increasing pattern in count of CD4^+^ among khat chewers, significant difference was not (P>0.05) observed from neirher chewer nor infected or healthy controls. On the other hand, cytotoxic-T lymphocytes or CD8^+^ count did not show significant differences (P>0.05) among different groups of the study participants (Fig 5).

## Discussion

Even though much emphasis has been given to genetic and environmental factors that determine host vulnerability to malaria and vector population, respectively [33], other factors associated with human behavior, and social, economic and cultural customs that might have a crucial role in burdening the disease were not assessed yet. However, in some malaria endemic areas, people might have frequent exposure to different substances for reasons such as stimulation, recreation or medicinal values. These factors might affect a host’s mechanisms of protection or increase vulnerability to some diseases by compromising its condition, or they might enhance the hosts means of protection and improve some clinical symptoms. Thus, khat leaf which is commonly chewed by most of malaria patients (57.38%) might be responsible for the suppression or expression of some common symptomes of malaria among khat chewers.

Most of the hyperpyrexia and its associated signs and vomiting symptoms have been largely attributed to production of various cytokines such as TNF-alpha produced in response to the parasite and toxin products released during rupture of infected RBCs [34]. Also, hemozoin released from infected RBCs (iRBCs) leading to the release of pro-inflammatory cytokines that in turn induce COX-2 (cyclooxygenase-2) up-regulating prostaglandins leading to the induction of fever [35, 36]. On the other hand, *in vitro* study on peripheral blood mononucleated cells (PBMCs) and *in vivo* study on animals model showed that khat has anti-inflammatory role on the pro-inflammatory cytokines responsible for induction of inflammation that`leads to fever during malaria infection by inhibiting their production and enhancing secretion of anti-inflammatory cytokines [37, 38].

The parasite load observed among khat users was also lower than the one among non-khat chewers. Even though anti-plasmodial activity of khat is not known yet, there are its reports on its antimicrobial activity against some human pathogens [39, 40], and it has also resistance-modifying potential [41]. The current report was in agreement with the practice documented among some endogenous people of East Africa and Meru tribe of Kenya who use khat for treatment of malaria [18–21]. Thus, the observed reduction in parasite burden among khat chewer malaria patients suggests the possible anti-plasmodial activity of the plant, but needs further confirmation.

There is a well established fact that changes that occur in liver function biomarkers induced by *P*. *falciparum* are the commonest form of malaria pathology [29]. It could occur due to alteration in blood flow through the organ as iRBCs adhere to endothelial cells, blocking the sinusoids and obstructing the intrahepatic blood flow [42]. Although there is equal likelihood for both chewers and non-khat chewers, higher risk of hepatic (jaundice) and renal dysfunctions was observed among khat chewer than non-khat chewer malaria patients. This malaria associated liver dysfunction is characterized by a rise in serum bilirubin along with a rise in serum GOT levels from mild abnormality to more than three times the upper limit of normal [43]. In this study, although more than three times the upper normal limit was observed in none, a significant increment of sGOT and sGPT levels was documented among khat chewer patients. Higher level of liver enzymes and other biomarkers such as lower level of albumin and higher level of total bilirubin observed in blood serum of khat chewer malaria patients could be accounted by their chronic exposure to khat. This is supported by different studies conducted on chronic khat chewers [44] and reports from animal models [45, 46]. Also, human and animal models exposed to khat showed the effect of the plant on kidney function [47]. According to these reports, chronic exposure to khat was negatively related to liver function. This finding revealed that in some areas where khat chewing is commonly practiced, khat might be one of the contributing factors to incidence of malaria associated hepatic and renal dysfunction, a fatal syndromes of *P*. *falciparum* parasite.

The renal impairment caused by khat could have further effects on the incidence of severe malaria complications through production of increased level of inflammation biomarker (uric acid). The significantly high level of CRP observed among khat chewer malaria patients is an indication for presence of excess uric acid (UA) in the bloods of the chewers. Source of this UA could be from the influence of khat on proper functioning of kidney, supposed to excrete it [48], and the *Plasmodium* parasite [49, 50]. Excess UA in blood facilitates production of inflammation cytokines and inflammatory molecules such as CRP, which will stimulate the production of IL-1β, IL-6 and TNF-α, that augments its pro-inflammatory properties and proliferation of the vascular smooth muscle cells [51, 52]. These all together facilitate the pathogenesis of cerebral malaria, mediated by excessive pro-inflammatory cytokines [53].

Although significant differences were not observed on some hematological tests among the two groups, as frequency (daily) and duration (chronic) of khat use increase, it is evidenced by the possibility of developing anemia and leucopenia among khat chewer malaria patients. Although there was no standard data available or generated on the status of patients’ renal failure, there were irregular complaints on urine retention problems among the khat chewer malaria patients.

Thrombocytopenia, an early sign of malaria infection, is among the most common malaria-associated hematological complications, with the incidence ranging from 40.5–85% in *P*. *vivax* and *P*. *falciparum* [54]. Its mechanism of occurrence was believed that low platelet counts in malaria might be caused by activation and/or apoptosis of platelets [55, 56], thus leading to its removal by the immune system, or immunity produce against the parasite antigen which could lead to adherence of the infected platelets in the spleen followed by phagocytosis by splenic macrophages [57–59]. A significant proportion of khat chewer malaria patients assessed in this study had lower level of platelet counts compared to non-khat chewer malaria patients. Thus malaria patients with a habit of khat chewing might be at higher risks of thrombocytopenia, lower level of platelets count, although the mechanism of reduction is not known. While the clinical symptoms being assessed, none of the participants complained of bleeding. In addition, the platelet counts of none of the patients was <50,000 cells/μL. All patients with lower platelet count were found between 100,000 and 150,000 cells/μL. Importantly, most of the patients whose platelet counts feel within this category were khat chewers. Thus, as this study was the first report for which we had no prior information, besides lack of complete blood cell count on spot, we could not further assess the details including the status of bleeding, kidney failure or liver impairment.

It is generally believed that khat chewing causes loss of appetite [60], and accounte for the lower body mass index of chewers. However, khat chewer malaria patients assessed in this study did not show significant reduction in body mass index (BMI). In fact, there are similar reports on BMI of khat chewers in different regions shows lack significant change or reduction [61, 62]. Thus, those severe malaria symptoms observed among khat chewer malaria patients might not be attributed to their poor nutritional status, one of the factors aggravating malaria infection among adolescents and adults in developing countries [63].

According to different literature and a very recent assessment made in the site of this study, people traditionally believe that khat has medicinal value for treatment of malaria [17–21]. However, except that it suppresses some early common symptoms of malaria such as vomiting, high grade-fever, diarrhea, and headache. which might be attributed to its anti-inflammatory nature, the medicinal value of khat was not observed. Instead, it has positive association with incidence of some life threatening severe malaria symptoms such as thrombocytopenia, hypoalbuminemia, hepatic and renal impairment and incidence of severe malaria complications as the number of admitted cases was higher among khat chewer malaria patients. The finding of this study might support the fact that khat has health and social problems reported earlier by different authors, like cognitive impairment, cardiovascular disorders, stomach ulcer, urine retention and gall bladder motility by relaxation of bladder wall and closure of internal sphincter, gastro-intestinal tract constipation and hemorrhage [64–67].

Khat use was positively associated with significant induction of antibodies secretion and CD4^+^ T-lymphocyte population during malaria infection. Different studies indicated that khat enhances *in vitro* secretion of anti-inflammatory cytokines and had anti-inflammatory role *in vivo* in rats, while suppressing secretion of pro-inflammatory cytokines [37, 38]. The presence of anti-inflammatory cytokines in the blood of khat user could facilitate differentiation of naïve helper T-lymphocytes into type 2 helper T cells (Th2) [68]. Then, Th2 further produce anti-inflammatory cytokines that facilitate induction of B-lymphocytes to develop into antibody secreting plasma cells and initiate isotype switching from IgM to other immunoglobulins or activate humeral immune response [68, 69]. Thus, the high titer antibodies (IgM and IgG calss/subclasses) observed among kaht chewer malaria patients could be attributed to khat that induces secretion of early anti-inflammatory mediatores.

Although both cellular and humeral immune responses are crucial for protection against malaria parasite, the antibody-dependent mechanisms play an important role in reduction of parasitaemia and suppression of clinical symptoms in humans [70]. In agreement to this, the elevated antibody titer measured among khat chewer and non-chewer malaria patients was negatively associated with parasite burden and manifestation of some severe malaria complications. The reduced manifestations of some early malaria symptoms such as lower parasitemia, vomiting, diarrhea and lower temperature could be attributed to the antibody secreted by the influence khat [70, 71] or the suppression of inflammatory cytokines responsible for pathogenesis of malaria pathologies [71].

Different studies suggested that among the four subclasses of IgG, IgG1 and IgG3 are the predominant subclasses produced in response to merozoite antigens [71–73]. IgG1 and IgG3 are cytophilic and T cell dependent, have high affinity for Fc receptors, and activate phagocytosis and activation of complement fixation [74]. They are also protective antibodies in endemic areas and associated with either lower parasitaemia or a lower risk of malaria attack [75, 76]. In agreement to earlier reports [75,76], the levels of these protective IgG subclasses (IgG1 and IgG3) observed among khat chewer *P*. *falciparum* patients was elevated and strongly associated with lower level of parasitemia and less incidence of severe clinical malaria pathologies.

T cells play a major role in the gaining and maintenance of protective immune response to malaria infection. Different studies from human and animals suggested that CD4^+^ cells are able to confer suppression of parasite growth and provide protection [77]. Helper T-cells, CD4^+^ regulates the immune response against malaria by proliferation and production of cytokines, and activation of B cells to produce parasite specific antibodies [77, 78]. In a study by Abiyu *et al* [79], chronic khat chewers were reported to have higher level of lymphocytes and CD4+ T-lymphocyte population. This study showed that khat further stimulates proliferation of CD4^+^ T-lymphocyte during malaria infection, as significantly higher count of this T- lymphocyte population was observed. Thus, besides direct involvement of CD4^+^ cells in parasite growth suppression and provide protection among khat users, it might support B lymphocytes activities for production of plasma antibodies.

## Conclusion

Even though some common malaria symptoms were not frequently observed among khat chewer malaria patients, khat might be an important risk factor for incidence of some severe malaria complications such as jaundice and renal impairment. Also, khat use was positively associated with high levels of antibodies titer and T- helper lymphocytes population. Moreover, the elevated levels of these antibodies were negatively correlated with parasite burden and manifestation of severe malaria pathologies.

## Supporting Information

S1 File Contains clinical data record form to be filled by physician or Nurses during data collection. (DOC)

S1 Table Contains correlation coefficients between antibodies among khat chewer malaria patients recruited at Halaba Kulito and Jimma Town Health Centers, Ethiopia. (DOC)

S2 Table Contains responses of khat chewer malaria patients on health consequences and medicinal value of khat. (DOC)

S3 Table Contains information on socio-demographic characteristics of khat chewer malaria patients. (DOC)

S4 Table Contains supportive data for uncomplicated and complicated malaria symptoms among khat chewer *P*. *falciparum* patients. (DOC)

S5 Table Contains data for severe malaria syndromes associated with frequency and duration of khat use. (DOC)
