# Supplementary material for: Incidence of Severe Malaria Syndromes and Status of Immune Responses among Khat Chewer Malaria Patients in Ethiopia
Source: PLoS One. 2015 Jul 14;10(7):e0131212. doi: 10.1371/journal.pone.0131212 (PMC4501669; doi:10.1371/journal.pone.0131212)
Supplement: S1 File — (DOC) [file pone.0131212.s001.doc]

**File S1** Clinical data record form to be filled by physician or Nurses during data collection.

- Code of patient’s _____________
- Sex ____
- Age____
- Weight _____
- Height _______
- Body temperature (^o^ C) ______

**Presence of severe malaria Symptoms will be assessed for all participants independently**

- Failure to feed ______
- Impaired conscious ______
- Weakness _______
- Vomiting _______
- Diarrhea ______
- Convulsion ________
- Fever ______
- Shock ______
- Headache_______
- Bleeding of gum or nose_______
- Comma_________
- Shock____________
- Respiratory distress__________

Splenomegaly__________

Hepatomegaly __________

Impaired consciousness______

Circulatory collapse ________

Rigors/chills__________

Sweat _________

Loss of appetite_______

Fatigue or weakness______

Nausea ________

Cough_________

Confusion __________

***Frequency of exposure to malaria infection***

How often you are exposed to malaria?

- Not at all _____
- Once/year ____
- Twice/year ____
- More than two/year ____

Did you get any medication during your malaria illness?

- Yes ____No_____

If your response is ‘No’ for the above question, what was the reason? Specify __________________________________________________

Do you have bed net in your home?

- Yes ____ No ______.

If your response to the above question is yes,

How many bed nets do you have? _________

How many of your family members sleep in bed net? ________

- All of them ________
- Only father & mother _________
- Only pregnant women _____________
- Only children _____________

For how long did you use? __________________

From where did you get? ____________________

iv. How often do you use?

- Always ________
- Sometime _______
- Not at all ________

Have you ever get insecticide spray?

- Yes ______ No_______

If your response is yes, in which year ________

***Exposure level to khat***

Have you ever chewed khat?

- Yes ______ No_____

If ‘yes’,

- At what age did you start khat chewing?
- When you were very young (<15 years) _____
- Between 15 and 20 years ________
- Between 20-25 years _____
  - 25 years _____

For how long did you chew khat? ___________________

How often do you chew chat?

- Once /day___________
- Once/week___________
- Twice/week___________
- ≥3 times/week___________

Are you chewing khat now? Yes ___________ No__________

What motivates you to chew khat?

________________________________________________

Do you have knowledge about health risk of khat?

- Yes ________ No ________

If your response is yes, what are they?

___________________________________________

Do you believe that khat chewing has health or social benefit?

- Yes ______ No______

If ‘yes’ describe the health benefit you think about khat.

_______________________________________________________

Mention social advantages can be obtained from chewing khat

________________________________________________________

What do you think about the current cost of khat? Can you afford it?

- Yes _______ No________________

If ‘No’ how do you get it?

_______________________________________________________

Did you try to stop khat chewing by any chance?

- Yes _____ No _______

If your response is yes, under what circumstance did you try? Specify _________________________________________________________

Did you manage to totally stop khat chewing? Yes _____ No_____

If your response is No, what was the reason? Specify, _________________________________

Besides khat chewing, what additional habits do you have?

- Alcohol drinking ________, Smoking _________, Both ________Any other__________

Have you ever admitted to any TB/HIV clinic for medication?

- Yes _______ No___________

If your response for the above question (5.3) is ‘yes’, what are they?
