# Supplementary material for: Incidence of Severe Malaria Syndromes and Status of Immune Responses among Khat Chewer Malaria Patients in Ethiopia
Source: PLoS One. 2015 Jul 14;10(7):e0131212. doi: 10.1371/journal.pone.0131212 (PMC4501669; doi:10.1371/journal.pone.0131212)
Supplement: S1 Table — (DOC) [file pone.0131212.s002.doc]

**S1 Table Correlation coefficients between antibodies among khat chewer malaria patients recruited at Halaba Kulito and Jimma Town Health Centers, Ethiopia**

|  |  | Antibody correlation | |  |  |  |
| --- | --- | --- | --- | --- | --- | --- |
| Coefficient r^†^, | IgG | IgM | IgG1 | IgG2 | IgG3 | IgG4 |
| IgG |  | 0.529^**^ | 0.587^**^ | 0.340^**^ | 0.240^**^ | 0.031 |
| IgM |  |  | 0.427^**^ | 0.229^*^ | 0.698^**^ | -0.056 |
| IgG1 |  |  |  | 0.122 | 0.394^**^ | 0.048 |
| IgG2 |  |  |  |  | 0.213^*^ | -0.015 |
| IgG3 |  |  |  |  |  | -0.017 |

^†^Spearman’s (r_s_) rank correlations were computed and statistical significance was considered when P <0.01^**^ or P <0.05^*^
