# Supplementary material for: Incidence of Severe Malaria Syndromes and Status of Immune Responses among Khat Chewer Malaria Patients in Ethiopia
Source: PLoS One. 2015 Jul 14;10(7):e0131212. doi: 10.1371/journal.pone.0131212 (PMC4501669; doi:10.1371/journal.pone.0131212)
Supplement: S2 Table — (DOC) [file pone.0131212.s003.doc]

S2 Table Responses of khat chewer malaria patients on health consequences and medicinal value of khat (n=210)

| **Health consequences** | | |
| --- | --- | --- |
| No | **Type of disease believed to be associated with khat chewing** | Respondents (%) |
| 1 | Brain disorder | 22 (10.5) |
| 2 | Carelessness | 189 (90) |
| 3 | Kidney problem | 54 (25.71) |
| 4 | Urinary tube blockage | 34 (16.2) |
| 5 | Stomachache | 157 (74.76) |
| 6 | Weight loss | 86 (40.95) |
| 7 | Loss of appetite | 168 (80) |
| 8 | Tooth decay | 178 (84.76) |
| 9 | Tooth discoloration | 205 (97.6) |
| 10 | Difficulty to sleep | 39 (18.57) |
| 11 | Dehydration | 35 (16.67) |
| **Respondents believed that khat chewing gives relief from the following disorders:** | | |
| 1 | Malaria | 147 (70) |
| 2 | Blood pressure | 84 (40) |
| 3 | Cold | 75 (35) |
| 4 | Depression | 105 (50) |
| 5 | Cough | 88 (42) |
| 6 | Stress | 80 (38) |
| 7 | Helminthes infection | 52 (24) |
| 8 | Diabetes | 63 (30) |
| 9 | Anemia | 55 (27) |
| 10 | Heart problem | 65 (31) |
| 11 | Diarrhea | 50 (24) |
| 12 | Headache | 25 (12) |
| 13 | Asthma | 31 (14.6) |
| 14 | Fever | 126 (60) |
| 15 | Others | 84 |
