# Supplementary material for: Incidence of Severe Malaria Syndromes and Status of Immune Responses among Khat Chewer Malaria Patients in Ethiopia
Source: PLoS One. 2015 Jul 14;10(7):e0131212. doi: 10.1371/journal.pone.0131212 (PMC4501669; doi:10.1371/journal.pone.0131212)
Supplement: S3 Table — (DOC) [file pone.0131212.s004.doc]

S3 Table Socio-demographic characteristics of khat chewer malaria patients

| Characteristics | No. Chewers (%) |
| --- | --- |
| **Age (year)**   - <20 - 20-30 - 31-40 - >40 |  |
|  | 30 (14.28) |
|  | 116 (55.23) |
|  | 38 (18.09) |
|  | 26 (12.38) |
| **Sex**   - Female - Male | 112 (53.33) |
|  | 98 (46.67 |
| **Education**   - Illiterate - Read and Write - Elementary complete - High school completed - Post high school | 42 (20) |
|  | 67 (31.9) |
|  | 52 (24.76) |
|  | 28 (13.33) |
|  | 21 (10) |
| **Marital status**   - Married - Single - Divorced - Widow | 151 (71.9) |
|  | 42 (20) |
|  | 12 (5.71) |
|  | 5 (2.38) |

| **Religion**   - Muslim - Protestant - Orthodox - Other | 185 (88.9) |
| --- | --- |
|  | 0 |
|  | 25 (11.9) |
|  | 0 |
| **Occupation**   - Gov. employee - Merchant - Farmer - Private - Daily laborer - Other | 32 (15.24) |
|  | 86 (40.9) |
|  | 12 (5.7) |
|  | 40 (19.04) |
|  | 16 (7.62) |
|  | 24 (11.43) |
