# Supplementary material for: Incidence of Severe Malaria Syndromes and Status of Immune Responses among Khat Chewer Malaria Patients in Ethiopia
Source: PLoS One. 2015 Jul 14;10(7):e0131212. doi: 10.1371/journal.pone.0131212 (PMC4501669; doi:10.1371/journal.pone.0131212)
Supplement: S4 Table — (DOC) [file pone.0131212.s005.doc]

**S4 Table. Supportive data for uncomplicated and complicated malaria symptoms among khat chewer *P. falciparum* patients** (NB: 1=yes, 0=no)

| **Patients Code** | **Khat chewing** | | **Frequency of use** | | | | | | | **Sex** | **Age** | **Smoking** | **Fever** | **Headache** | **Vomiting** | **Diarrhea** | **Bed net** | **Hyperpyrexia** | **Prostration** | **Jaundice** | **Impaired consciousness** | **Respiratory distress** | | **Multiple convulsion** | | **Abnormal bleeding** | | **Hyper-parasitemia** | | **Hypoglycemia** | | **Severe anemia** | |
| --- | --- | --- | --- | --- | --- | --- | --- | --- | --- | --- | --- | --- | --- | --- | --- | --- | --- | --- | --- | --- | --- | --- | --- | --- | --- | --- | --- | --- | --- | --- | --- | --- | --- |
| H001 | 1 |  | 1 | | | | | | | M | 27 | 0 | 1 | 1 | 0 | 0 | 0 | 0 | 0 | 0 | 0 | 0 | | 0 | | 0 | | 0 | | 0 | | 0 | |
| H002 | 0 |  | 0 | | | | | | | F | 40 | 0 | 1 | 1 | 1 | 1 | 0 | 0 | 1 | 0 | 0 | 0 | | 0 | | 0 | | 0 | | 0 | | 0 | |
| H003 | 1 |  | 1 | | | | | | | M | 25 | 0 | 1 | 1 | 0 | 0 | 1 | 0 | 0 | 1 | 0 | 0 | | 0 | | 0 | | 1 | | 0 | | 0 | |
| H004 | 0 |  | 0 | | | | | | | F | 55 | 0 | 1 | 1 | 0 | 0 | 1 | 0 | 0 | 0 | 0 | 0 | | 0 | | 0 | | 0 | | 0 | | 0 | |
| H005 | 0 |  | 0 | | | | | | | F | 27 | 0 | 1 | 1 | 1 | 1 | 1 | 0 | 1 | 1 | 1 | 1 | | 0 | | 0 | | 1 | | 0 | | 0 | |
| H006 | 0 |  | 0 | | | | | | | F | 14 | 0 | 1 | 1 | 0 | 0 | 1 | 1 | 1 | 0 | 0 | 0 | | 0 | | 0 | | 0 | | 0 | | 0 | |
| H007 | 0 |  | 0 | | | | | | | M | 23 | 0 | 1 | 1 | 1 | 0 | 0 | 0 | 0 | 0 | 0 | 0 | | 0 | | 0 | | 0 | | 0 | | 0 | |
| H008 | 1 |  | 2 | | | | | | | M | 26 | 0 | 1 | 1 | 0 | 0 | 1 | 0 | 0 | 0 | 1 | 0 | | 0 | | 0 | | 0 | | 0 | | 0 | |
| H009 | 0 |  | 0 | | | | | | | F | 14 | 0 | 1 | 1 | 0 | 0 | 0 | 0 | 1 | 0 | 0 | 0 | | 0 | | 0 | | 1 | | 0 | | 0 | |
| H010 | 0 |  | 0 | | | | | | | M | 25 | 0 | 0 | 1 | 0 | 0 | 0 | 0 | 0 | 1 | 1 | 1 | | 0 | | 0 | | 0 | | 0 | | 0 | |
| H011 | 1 |  | 1 | | | | | | | M | 23 | 0 | 1 | 1 | 0 | 0 | 1 | 0 | 0 | 0 | 1 | 0 | | 0 | | 0 | | 0 | | 0 | | 0 | |
| H012 | 0 |  | 0 | | | | | | | M | 30 | 0 | 1 | 0 | 0 | 0 | 1 | 0 | 0 | 0 | 0 | 0 | | 0 | | 0 | | 0 | | 0 | | 0 | |
| H013 | 1 |  | 3 | | | | | | | F | 44 | 0 | 1 | 1 | 0 | 0 | 1 | 0 | 1 | 0 | 1 | 1 | | 0 | | 0 | | 1 | | 0 | | 0 | |
| H014 | 0 |  | 0 | | | | | | | F | 25 | 0 | 1 | 1 | 1 | 0 | 1 | 1 | 0 | 0 | 0 | 0 | | 0 | | 0 | | 0 | | 0 | | 0 | |
| H015 | 0 |  | 0 | | | | | | | M | 16 | 0 | 1 | 1 | 1 | 1 | 1 | 0 | 1 | 0 | 0 | 0 | | 0 | | 0 | | 0 | | 0 | | 0 | |
| H016 | 1 |  | | | | | | 2 | | F | 29 | 0 | 1 | 1 | 0 | 0 | 0 | 0 | 0 | 0 | 0 | | 0 | | 0 | | 0 | | 0 | | 0 | | 0 |
| H017 | 1 |  | | | | | | 1 | | M | 25 | 0 | 1 | 1 | 1 | 1 | 1 | 0 | 0 | 1 | 0 | | 0 | | 0 | | 0 | | 0 | | 0 | | 0 |
| H018 | 0 |  | | | | | | 0 | | M | 28 | 0 | 1 | 1 | 1 | 0 | 0 | 1 | 1 | 0 | 1 | | 1 | | 0 | | 0 | | 0 | | 0 | | 0 |
| H019 | 1 |  | | | | | | 1 | | M | 15 | 0 | 1 | 1 | 0 | 0 | 1 | 0 | 1 | 0 | 0 | | 0 | | 0 | | 0 | | 1 | | 0 | | 0 |
| H020 | 1 |  | | | | | | 3 | | F | 35 | 0 | 1 | 1 | 1 | 0 | 1 | 0 | 0 | 0 | 0 | | 0 | | 0 | | 0 | | 0 | | 0 | | 0 |
| H021 | 0 |  | | | | | | 0 | | M | 41 | 0 | 1 | 1 | 1 | 0 | 0 | 0 | 1 | 0 | 0 | | 0 | | 0 | | 0 | | 0 | | 0 | | 0 |
| H022 | 1 |  | | | | | | 1 | | M | 25 | 0 | 1 | 1 | 0 | 0 | 1 | 0 | 0 | 0 | 0 | | 0 | | 0 | | 0 | | 0 | | 0 | | 0 |
| H023 | 0 |  | | | | | | 0 | | F | 50 | 0 | 0 | 1 | 1 | 0 | 1 | 0 | 1 | 0 | 0 | | 0 | | 0 | | 0 | | 0 | | 0 | | 0 |
| H024 | 1 |  | | | | | | 1 | | M | 52 | 0 | 1 | 1 | 0 | 0 | 1 | 0 | 0 | 0 | 0 | | 0 | | 0 | | 0 | | 0 | | 0 | | 0 |
| H025 | 1 |  | | | | | | 1 | | F | 17 | 0 | 1 | 1 | 1 | 0 | 1 | 0 | 0 | 0 | 1 | | 1 | | 0 | | 0 | | 1 | | 0 | | 0 |
| H026 | 1 |  | | | | | | 2 | | M | 24 | 0 | 1 | 1 | 1 | 0 | 1 | 0 | 0 | 1 | 0 | | 0 | | 0 | | 0 | | 0 | | 0 | | 0 |
| H027 | 0 |  | | | | | | 0 | | F | 18 | 0 | 1 | 1 | 0 | 0 | 0 | 0 | 1 | 0 | 0 | | 0 | | 0 | | 0 | | 0 | | 0 | | 0 |
| H028 | 1 |  | | | | | | 2 | | F | 16 | 0 | 1 | 1 | 0 | 0 | 1 | 0 | 0 | 0 | 1 | | 0 | | 0 | | 0 | | 0 | | 0 | | 0 |
| H029 | 0 |  | | | | | | 0 | | F | 25 | 0 | 1 | 1 | 0 | 0 | 1 | 0 | 0 | 0 | 0 | | 0 | | 0 | | 0 | | 0 | | 0 | | 0 |
| H030 | 1 |  | | | | | | 3 | | F | 45 | 0 | 1 | 1 | 1 | 0 | 0 | 0 | 0 | 0 | 0 | | 0 | | 0 | | 0 | | 0 | | 0 | | 0 |
| H031 | 1 |  | | | | | | 1 | | M | 28 | 0 | 1 | 1 | 0 | 0 | 1 | 0 | 0 | 1 | 0 | | 0 | | 0 | | 0 | | 0 | | 0 | | 0 |
| H032 | 0 |  | | | | | | 0 | | F | 27 | 0 | 1 | 1 | 0 | 0 | 1 | 1 | 1 | 0 | 0 | | 0 | | 0 | | 0 | | 0 | | 0 | | 0 |
| H033 | 0 |  | | | | | | 0 | | M | 50 | 0 | 1 | 1 | 0 | 0 | 1 | 0 | 1 | 0 | 0 | | 0 | | 0 | | 0 | | 0 | | 0 | | 0 |
| H034 | 0 |  | | | | | | 0 | | F | 15 | 0 | 1 | 1 | 1 | 1 | 1 | 0 | 1 | 1 | 0 | | 0 | | 0 | | 0 | | 0 | | 0 | | 0 |
| H035 | 1 |  | | | | | | 1 | | M | 25 | 0 | 1 | 1 | 0 | 0 | 0 | 0 | 0 | 0 | 0 | | 0 | | 0 | | 0 | | 0 | | 0 | | 0 |
| H036 | 1 |  | | | | | | 1 | | M | 37 | 0 | 0 | 1 | 1 | 0 | 1 | 0 | 0 | 0 | 1 | | 1 | | 0 | | 0 | | 0 | | 0 | | 0 |
| H037 | 1 |  | | | | | | 3 | | F | 18 | 0 | 1 | 1 | 1 | 0 | 1 | 0 | 0 | 0 | 1 | | 0 | | 0 | | 0 | | 0 | | 0 | | 0 |
| H038 | 0 |  | | | | | | 0 | | F | 14 | 0 | 1 | 1 | 0 | 0 | 1 | 0 | 1 | 0 | 0 | | 0 | | 0 | | 0 | | 0 | | 0 | | 0 |
| H039 | 1 |  | | | | | | 1 | | F | 35 | 0 | 1 | 1 | 1 | 0 | 1 | 0 | 0 | 0 | 0 | | 0 | | 0 | | 0 | | 0 | | 0 | | 0 |
| H040 | 1 |  | | | | | | 1 | | M | 34 | 1 | 1 | 1 | 1 | 0 | 1 | 0 | 1 | 0 | 0 | | 0 | | 0 | | 0 | | 0 | | 0 | | 0 |
| H041 | 0 |  | | | | | | 0 | | M | 50 | 0 | 1 | 1 | 0 | 0 | 0 | 0 | 0 | 0 | 0 | | 0 | | 0 | | 0 | | 0 | | 0 | | 0 |
| H042 | 0 |  | | 0 | | | | | | M | 41 | 1 | 1 | 1 | 0 | 0 | 1 | 1 | 0 | 0 | 0 | | 0 | | 0 | | 0 | | 0 | | 0 | | 0 |
| H043 | 1 |  | | | | 1 | | | | F | 27 | 0 | 1 | 1 | 1 | 0 | 1 | 0 | 0 | 0 | 0 | | 0 | | 0 | | 0 | | 0 | | 0 | | 0 |
| H044 | 1 |  | | | | 1 | | | | F | 40 | 0 | 0 | 1 | 1 | 0 | 0 | 0 | 0 | 0 | 1 | | 0 | | 0 | | 0 | | 0 | | 0 | | 0 |
| H045 | 1 |  | | | | 2 | | | | M | 25 | 0 | 1 | 1 | 0 | 0 | 0 | 0 | 0 | 0 | 0 | | 0 | | 0 | | 0 | | 0 | | 0 | | 0 |
| H046 | 1 |  | | | | 1 | | | | F | 43 | 0 | 0 | 1 | 1 | 0 | 0 | 0 | 1 | 1 | 1 | | 1 | | 0 | | 0 | | 0 | | 0 | | 0 |
| H047 | 1 |  | | | | 1 | | | | M | 25 | 1 | 1 | 1 | 1 | 1 | 1 | 0 | 0 | 1 | 1 | | 1 | | 0 | | 0 | | 0 | | 0 | | 0 |
| H048 | 1 |  | | | | 1 | | | | M | 24 | 0 | 1 | 1 | 0 | 0 | 1 | 0 | 0 | 0 | 0 | | 0 | | 0 | | 0 | | 0 | | 0 | | 0 |
| H049 | 1 |  | | | | 3 | | | | F | 14 | 0 | 1 | 1 | 0 | 0 | 1 | 0 | 0 | 0 | 1 | | 0 | | 0 | | 0 | | 0 | | 0 | | 0 |
| H050 | 0 |  | | | | 0 | | | | M | 14 | 0 | 1 | 1 | 1 | 0 | 0 | 1 | 1 | 0 | 0 | | 0 | | 0 | | 0 | | 0 | | 0 | | 0 |
| H051 | 0 |  | | | | 0 | | | | F | 30 | 0 | 1 | 1 | 0 | 0 | 1 | 0 | 0 | 1 | 1 | | 1 | | 0 | | 0 | | 0 | | 0 | | 0 |
| H052 | 1 |  | | | | 1 | | | | M | 49 | 1 | 1 | 1 | 1 | 1 | 1 | 0 | 0 | 0 | 1 | | 0 | | 0 | | 0 | | 0 | | 0 | | 0 |
| H053 | 0 |  | | | | 0 | | | | F | 32 | 0 | 1 | 1 | 1 | 0 | 1 | 0 | 1 | 1 | 0 | | 0 | | 0 | | 0 | | 0 | | 0 | | 0 |
| H054 | 0 |  | | | | 0 | | | | F | 18 | 0 | 1 | 1 | 0 | 0 | 0 | 0 | 0 | 0 | 0 | | 0 | | 0 | | 0 | | 0 | | 0 | | 0 |
| H055 | 0 |  | | | | 0 | | | | M | 48 | 0 | 1 | 1 | 1 | 1 | 1 | 0 | 0 | 0 | 0 | | 0 | | 0 | | 0 | | 0 | | 0 | | 0 |
| H056 | 0 |  | | | | 0 | | | | M | 27 | 0 | 1 | 1 | 1 | 0 | 1 | 0 | 1 | 0 | 1 | | 1 | | 0 | | 0 | | 0 | | 0 | | 0 |
| H057 | 0 |  | | | | 0 | | | | F | 26 | 0 | 1 | 1 | 0 | 0 | 1 | 0 | 1 | 0 | 0 | | 0 | | 0 | | 0 | | 0 | | 0 | | 0 |
| H058 | 1 |  | | | | 1 | | | | M | 36 | 0 | 1 | 1 | 1 | 1 | 1 | 0 | 0 | 0 | 1 | | 1 | | 0 | | 0 | | 0 | | 0 | | 0 |
| H059 | 1 |  | | | | 1 | | | | M | 23 | 0 | 1 | 1 | 0 | 0 | 0 | 0 | 0 | 0 | 1 | | 1 | | 0 | | 0 | | 0 | | 0 | | 0 |
| H060 | 1 |  | | | | 1 | | | | M | 19 | 0 | 1 | 1 | 1 | 0 | 1 | 0 | 0 | 0 | 0 | | 0 | | 0 | | 0 | | 0 | | 0 | | 0 |
| H061 | 1 |  | | | | 2 | | | | M | 20 | 0 | 0 | 0 | 0 | 0 | 0 | 0 | 0 | 0 | 0 | | 0 | | 0 | | 0 | | 0 | | 0 | | 0 |
| H062 | 1 |  | | | | 3 | | | | F | 22 | 0 | 1 | 0 | 0 | 0 | 1 | 0 | 1 | 0 | 0 | | 0 | | 0 | | 0 | | 1 | | 0 | | 0 |
| H063 | 1 |  | | | | 1 | | | | M | 30 | 1 | 1 | 0 | 1 | 0 | 1 | 0 | 0 | 0 | 1 | | 1 | | 0 | | 0 | | 1 | | 0 | | 0 |
| H064 | 1 |  | | | | 1 | | | | F | 30 | 0 | 1 | 0 | 1 | 0 | 0 | 0 | 0 | 0 | 0 | | 0 | | 0 | | 0 | | 0 | | 0 | | 0 |
| H065 | 1 |  | | | | 1 | | | | F | 20 | 0 | 0 | 0 | 1 | 0 | 1 | 0 | 0 | 1 | 0 | | 0 | | 0 | | 0 | | 0 | | 0 | | 0 |
| H066 | 0 |  | | | | 0 | | | | M | 29 | 0 | 1 | 0 | 0 | 0 | 0 | 0 | 0 | 0 | 0 | | 0 | | 0 | | 0 | | 0 | | 0 | | 0 |
| H067 | 1 |  | | | | 2 | | | | M | 60 | 0 | 0 | 0 | 1 | 0 | 0 | 0 | 0 | 0 | 0 | | 0 | | 0 | | 0 | | 0 | | 0 | | 0 |
| H068 | 0 |  | 0 | | | | | | | F | 20 | 0 | 1 | 0 | 1 | 0 | 1 | 0 | 0 | 0 | 0 | | 0 | | 0 | | 0 | | 0 | | 0 | | 0 |
| H069 | 1 |  | 1 | | | | | | | F | 35 | 0 | 0 | 0 | 1 | 0 | 1 | 0 | 0 | 0 | 0 | | 0 | | 0 | | 0 | | 0 | | 0 | | 0 |
| H070 | 0 |  | 0 | | | | | | | F | 20 | 0 | 1 | 0 | 1 | 0 | 1 | 0 | 0 | 0 | 0 | | 0 | | 0 | | 0 | | 0 | | 0 | | 0 |
| H071 | 1 |  | 1 | | | | | | | F | 25 | 0 | 0 | 0 | 1 | 0 | 1 | 0 | 0 | 0 | 1 | | 1 | | 0 | | 0 | | 0 | | 0 | | 0 |
| H072 | 1 |  | 1 | | | | | | | M | 38 | 0 | 0 | 1 | 0 | 0 | 1 | 0 | 0 | 0 | 1 | | 1 | | 0 | | 0 | | 0 | | 0 | | 0 |
| H073 | 0 |  | 0 | | | | | | | M | 29 |  | 1 | 1 | 1 | 1 | 1 | 0 | 0 | 0 | 0 | | 0 | | 0 | | 0 | | 0 | | 0 | | 0 |
| H074 | 1 |  | 1 | | | | | | | M | 30 | 0 | 1 | 1 | 0 | 0 | 1 | 0 | 1 | 0 | 0 | | 0 | | 0 | | 0 | | 0 | | 0 | | 0 |
| H075 | 0 |  | 0 | | | | | | | M | 34 | 0 | 1 | 1 | 1 | 0 | 0 | 0 | 0 | 0 | 0 | | 0 | | 0 | | 0 | | 0 | | 0 | | 0 |
| H076 | 1 |  | 1 | | | | | | | M | 23 | 0 | 0 | 1 | 0 | 0 | 1 | 0 | 0 | 0 | 0 | | 0 | | 0 | | 0 | | 0 | | 0 | | 0 |
| H077 | 0 |  | 0 | | | | | | | M | 35 | 0 | 1 | 1 | 1 | 0 | 1 | 0 | 0 | 0 | 0 | | 0 | | 0 | | 0 | | 0 | | 0 | | 0 |
| H078 | 1 |  | 2 | | | | | | | M | 45 | 0 | 0 | 1 | 0 | 1 | 1 | 0 | 0 | 1 | 1 | | 1 | | 0 | | 0 | | 0 | | 0 | | 0 |
| H079 | 0 |  | 0 | | | | | | | F | 29 | 0 | 1 | 1 | 0 | 0 | 1 | 1 | 1 | 0 | 0 | | 0 | | 0 | | 0 | | 0 | | 0 | | 0 |
| H080 | 1 |  | 3 | | | | | | | M | 20 | 0 | 1 | 1 | 0 | 0 | 1 | 0 | 0 | 0 | 0 | | 0 | | 0 | | 0 | | 0 | | 0 | | 0 |
| H081 | 0 |  | 0 | | | | | | | F | 27 | 0 | 1 | 1 | 0 | 0 | 1 | 0 | 0 | 0 | 0 | | 0 | | 0 | | 0 | | 0 | | 0 | | 0 |
| H082 | 1 |  | 1 | | | | | | | F | 14 | 0 | 1 | 1 | 0 | 0 | 1 | 0 | 0 | 0 | 0 | | 0 | | 0 | | 0 | | 0 | | 0 | | 0 |
| H083 | 0 |  | 0 | | | | | | | M | 54 | 0 | 1 | 1 | 1 | 0 | 1 | 0 | 0 | 0 | 0 | | 0 | | 0 | | 0 | | 0 | | 0 | | 0 |
| H084 | 0 |  | 0 | | | | | | | F | 21 | 0 | 1 | 1 | 1 | 0 | 0 | 0 | 1 | 0 | 1 | | 1 | | 0 | | 0 | | 0 | | 0 | | 0 |
| H085 | 0 |  | 0 | | | | | | | M | 30 | 0 | 1 | 1 | 1 | 0 | 0 | 0 | 1 | 0 | 0 | | 0 | | 0 | | 0 | | 0 | | 0 | | 0 |
| H086 | 0 |  | 0 | | | | | | | F | 40 | 0 | 1 | 1 | 1 | 0 | 1 | 0 | 0 | 0 | 0 | | 0 | | 0 | | 0 | | 0 | | 0 | | 0 |
| H087 | 1 |  | 1 | | | | | | | F | 55 | 0 | 0 | 0 | 0 | 0 | 0 | 0 | 0 | 1 | 1 | | 1 | | 0 | | 0 | | 1 | | 0 | | 0 |
| H088 | 0 |  | 0 | | | | | | | M | 16 | 0 | 1 | 1 | 1 | 1 | 0 | 0 | 0 | 0 | 0 | | 0 | | 0 | | 0 | | 0 | | 0 | | 0 |
| H089 | 0 |  | 0 | | | | | | | F | 29 | 0 | 1 | 1 | 1 | 0 | 0 | 0 | 1 | 0 | 1 | | 1 | | 0 | | 0 | | 0 | | 0 | | 0 |
| H090 | 0 |  | 0 | | | | | | | M | 25 | 0 | 1 | 1 | 1 | 0 | 1 | 0 | 1 | 0 | 0 | | 0 | | 0 | | 0 | | 0 | | 0 | | 0 |
| H091 | 1 |  | 1 | | | | | | | M | 28 | 0 | 0 | 1 | 0 | 0 | 0 | 0 | 0 | 0 | 0 | | 0 | | 0 | | 0 | | 0 | | 0 | | 0 |
| H092 | 0 |  | 0 | | | | | | | M | 15 | 0 | 1 | 1 | 1 | 1 | 1 | 0 | 1 | 0 | 0 | | 0 | | 0 | | 0 | | 0 | | 0 | | 0 |
| H093 | 0 |  | 0 | | | | | | | F | 35 | 0 | 1 | 0 | 1 | 1 | 0 | 0 | 1 | 0 | 0 | | 0 | | 0 | | 0 | | 0 | | 0 | | 0 |
| H094 | 1 |  | | | | | | | 1 | M | 30 | 0 | 0 | 1 | 1 | 0 | 1 | 0 | 0 | 0 | 0 | | 0 | | 0 | | 0 | | 0 | | 0 | | 0 |
| H095 | 1 |  | | | | | | | 3 | M | 41 | 0 | 1 | 1 | 0 | 0 | 1 | 0 | 0 | 0 | 0 | | 0 | | 0 | | 0 | | 0 | | 0 | | 0 |
| H096 | 0 |  | | | | | | | 0 | F | 50 | 0 | 1 | 1 | 1 | 0 | 0 | 1 | 0 | 0 | 0 | | 0 | | 0 | | 0 | | 0 | | 0 | | 0 |
| H097 | 0 |  | | | | | | | 0 | M | 52 | 0 | 1 | 1 | 1 | 1 | 1 | 0 | 1 | 0 | 0 | | 0 | | 0 | | 0 | | 0 | | 0 | | 0 |
| H098 | 1 |  | | | | | | | 3 | M | 17 | 0 | 0 | 1 | 1 | 0 | 1 | 0 | 0 | 0 | 0 | | 0 | | 0 | | 0 | | 0 | | 0 | | 0 |
| H099 | 0 |  | | | | | | | 0 | M | 28 | 0 | 1 | 1 | 1 | 1 | 1 | 0 | 0 | 0 | 0 | | 0 | | 0 | | 0 | | 0 | | 0 | | 0 |
| H100 | 0 |  | | | | | | | 0 | F | 18 | 0 | 1 | 1 | 0 | 1 | 1 | 0 | 0 | 1 | 1 | | 1 | | 0 | | 0 | | 0 | | 0 | | 0 |
| H101 | 1 |  | | | | | | | 1 | F | 34 | 0 | 1 | 1 | 0 | 0 | 0 | 0 | 0 | 0 | 0 | | 0 | | 0 | | 0 | | 0 | | 0 | | 0 |
| H102 | 1 |  | | | | | | | 1 | F | 25 | 0 | 1 | 1 | 1 | 0 | 1 | 0 | 0 | 0 | 0 | | 0 | | 0 | | 0 | | 0 | | 0 | | 0 |
| H103 | 1 |  | | | | | | | 2 | F | 30 | 0 | 1 | 1 | 1 | 0 | 1 | 0 | 1 | 1 | 1 | | 1 | | 0 | | 0 | | 0 | | 0 | | 0 |
| H104 | 1 |  | | | | | | | 2 | M | 20 | 0 | 0 | 1 | 1 | 0 | 0 | 0 | 0 | 0 | 0 | | 0 | | 0 | | 0 | | 0 | | 0 | | 0 |
| H105 | 0 |  | | | | | | | 0 | F | 27 | 0 | 1 | 1 | 1 | 0 | 0 | 0 | 1 | 0 | 0 | | 0 | | 0 | | 0 | | 0 | | 0 | | 0 |
| H106 | 1 |  | | | | | | | 1 | F | 50 | 0 | 1 | 1 | 0 | 0 | 0 | 0 | 0 | 0 | 0 | | 0 | | 0 | | 0 | | 1 | | 0 | | 0 |
| H107 | 1 |  | | | | | | | 1 | F | 15 | 0 | 0 | 1 | 0 | 0 | 1 | 0 | 0 | 0 | 1 | | 1 | | 0 | | 0 | | 0 | | 0 | | 0 |
| H108 | 1 |  | | | | | | | 3 | M | 28 | 0 | 1 | 1 | 1 | 0 | 1 | 0 | 0 | 0 | 0 | | 0 | | 0 | | 0 | | 0 | | 0 | | 0 |
| H109 | 1 |  | | | | | | | 1 | M | 37 | 0 | 1 | 1 | 0 | 0 | 0 | 0 | 0 | 0 | 1 | | 1 | | 0 | | 0 | | 0 | | 0 | | 0 |
| H110 | 1 |  | | | | | | | 3 | F | 18 | 0 | 1 | 1 | 0 | 1 | 1 | 0 | 0 | 0 | 1 | | 1 | | 0 | | 0 | | 1 | | 0 | | 0 |
| H111 | 1 |  | | | | | | | 3 | F | 14 | 0 | 1 | 1 | 0 | 0 | 0 | 0 | 1 | 0 | 0 | | 0 | | 0 | | 0 | | 0 | | 0 | | 0 |
| H112 | 0 |  | | | | | | | 0 | F | 35 | 0 | 1 | 1 | 1 | 1 | 1 | 0 | 0 | 0 | 0 | | 0 | | 0 | | 0 | | 0 | | 0 | | 0 |
| H113 | 0 |  | | | | | | | 0 | M | 34 | 0 | 1 | 1 | 1 | 0 | 0 | 0 | 0 | 0 | 0 | | 0 | | 0 | | 0 | | 1 | | 0 | | 0 |
| H114 | 1 |  | | | | | | | 1 | M | 50 | 0 | 0 | 1 | 1 | 0 | 1 | 0 | 0 | 0 | 0 | | 0 | | 0 | | 0 | | 0 | | 0 | | 0 |
| H115 | 0 |  | | | | | | | 0 | M | 41 | 0 | 1 | 1 | 1 | 0 | 0 | 0 | 0 | 0 | 0 | | 0 | | 0 | | 0 | | 0 | | 0 | | 0 |
| H116 | 1 |  | | | | | | | 2 | F | 27 | 0 | 1 | 1 | 1 | 1 | 0 | 0 | 1 | 0 | 0 | | 0 | | 0 | | 0 | | 0 | | 0 | | 0 |
| H117 | 0 |  | | | | | | | 0 | F | 40 | 0 | 1 | 1 | 0 | 1 | 1 | 0 | 1 | 0 | 0 | | 0 | | 0 | | 0 | | 0 | | 0 | | 0 |
| H118 | 0 |  | | | | | | | 0 | M | 25 | 0 | 1 | 1 | 1 | 0 | 1 | 0 | 0 | 0 | 1 | | 1 | | 0 | | 0 | | 0 | | 0 | | 0 |
| H119 | 1 |  | | | | | | | 3 | F | 30 | 0 | 0 | 1 | 1 | 0 | 1 | 0 | 0 | 0 | 0 | | 0 | | 0 | | 0 | | 0 | | 0 | | 0 |
| H120 | 1 |  | | | | 1 | | | | M | 25 | 0 | 1 | 1 | 0 | 0 | 0 | 0 | 0 | 0 | 0 | | 0 | | 0 | | 0 | | 0 | | 0 | | 0 |
| H121 | 1 |  | | | | 1 | | | | M | 20 | 0 | 1 | 1 | 0 | 0 | 1 | 0 | 1 | 0 | 0 | | 0 | | 0 | | 0 | | 1 | | 0 | | 0 |
| H122 | 0 |  | | | | 0 | | | | F | 14 | 0 | 1 | 1 | 1 | 1 | 0 | 1 | 0 | 0 | 0 | | 0 | | 0 | | 0 | | 0 | | 0 | | 0 |
| H123 | 1 |  | | | | 1 | | | | M | 29 | 0 | 0 | 0 | 1 | 0 | 1 | 0 | 0 | 0 | 0 | | 0 | | 0 | | 0 | | 0 | | 0 | | 0 |
| H124 | 1 |  | | | | 3 | | | | F | 42 | 0 | 1 | 0 | 1 | 0 | 1 | 0 | 0 | 0 | 1 | | 1 | | 0 | | 0 | | 0 | | 0 | | 0 |
| H125 | 1 |  | | | | 2 | | | | M | 30 | 1 | 1 | 1 | 0 | 0 | 0 | 0 | 1 | 0 | 0 | | 0 | | 0 | | 0 | | 0 | | 0 | | 0 |
| H126 | 1 |  | | | | 1 | | | | F | 30 | 0 | 0 | 1 | 0 | 1 | 1 | 0 | 0 | 0 | 0 | | 0 | | 0 | | 0 | | 0 | | 0 | | 0 |
| H127 | 0 |  | | | | 0 | | | | F | 18 | 0 | 1 | 1 | 0 | 1 | 1 | 0 | 0 | 0 | 0 | | 0 | | 0 | | 0 | | 0 | | 0 | | 0 |
| H128 | 1 |  | | | | 3 | | | | M | 14 | 0 | 0 | 1 | 1 | 0 | 0 | 0 | 1 | 0 | 0 | | 0 | | 0 | | 0 | | 0 | | 0 | | 0 |
| H129 | 1 |  | | | | 3 | | | | M | 27 | 0 | 1 | 1 | 0 | 0 | 1 | 0 | 0 | 0 | 0 | | 0 | | 0 | | 0 | | 0 | | 0 | | 0 |
| H130 | 0 |  | | | | 0 | | | | F | 25 | 0 | 1 | 1 | 1 | 1 | 1 | 0 | 1 | 0 | 0 | | 0 | | 0 | | 0 | | 0 | | 0 | | 0 |
| H131 | 0 |  | | | | 0 | | | | M | 36 | 0 | 1 | 1 | 1 | 1 | 1 | 0 | 1 | 0 | 0 | | 0 | | 0 | | 0 | | 1 | | 0 | | 0 |
| H132 | 0 |  | | | | 0 | | | | M | 23 | 0 | 1 | 1 | 1 | 0 | 1 | 1 | 1 | 0 | 1 | | 1 | | 0 | | 0 | | 0 | | 0 | | 0 |
| H133 | 0 |  | | | | 0 | | | | M | 19 | 0 | 1 | 1 | 1 | 0 | 1 | 0 | 0 | 0 | 0 | | 0 | | 0 | | 0 | | 0 | | 0 | | 0 |
| H134 | 1 |  | | | | 2 | | | | M | 20 | 0 | 1 | 1 | 0 | 0 | 0 | 0 | 0 | 0 | 0 | | 0 | | 0 | | 0 | | 0 | | 0 | | 0 |
| H135 | 1 |  | | | | 1 | | | | F | 22 | 0 | 0 | 1 | 0 | 1 | 1 | 0 | 0 | 0 | 0 | | 0 | | 0 | | 0 | | 0 | | 0 | | 0 |
| H136 | 1 |  | | | | 1 | | | | M | 30 | 0 | 1 | 1 | 0 | 1 | 1 | 0 | 0 | 0 | 1 | | 1 | | 0 | | 0 | | 0 | | 0 | | 0 |
| H137 | 0 |  | | | | 0 | | | | F | 30 | 0 | 1 | 1 | 1 | 0 | 1 | 0 | 0 | 0 | 0 | | 0 | | 0 | | 0 | | 0 | | 0 | | 0 |
| H138 | 1 |  | | | | 1 | | | | F | 20 | 0 | 1 | 1 | 1 | 0 | 0 | 0 | 0 | 0 | 0 | | 0 | | 0 | | 0 | | 0 | | 0 | | 0 |
| H139 | 1 |  | | | | 1 | | | | M | 25 | 0 | 1 | 1 | 0 | 0 | 0 | 0 | 0 | 0 | 0 | | 0 | | 0 | | 0 | | 0 | | 0 | | 0 |
| H140 | 1 |  | | | | 2 | | | | M | 60 | 0 | 1 | 1 | 0 | 0 | 1 | 0 | 0 | 0 | 0 | | 0 | | 0 | | 0 | | 0 | | 0 | | 0 |
| H141 | 0 |  | | | | 0 | | | | F | 20 | 0 | 1 | 1 | 1 | 1 | 1 | 0 | 1 | 0 | 0 | | 0 | | 0 | | 0 | | 0 | | 0 | | 0 |
| H142 | 1 |  | | | | 3 | | | | F | 35 | 0 | 1 | 1 | 1 | 0 | 1 | 0 | 0 | 0 | 0 | | 0 | | 0 | | 0 | | 0 | | 0 | | 0 |
| H143 | 1 |  | | | | 1 | | | | F | 30 | 0 | 1 | 1 | 1 | 0 | 1 | 0 | 0 | 0 | 0 | | 0 | | 0 | | 0 | | 0 | | 0 | | 0 |
| H144 | 1 |  | | | | 2 | | | | M | 20 | 0 | 1 | 1 | 0 | 0 | 0 | 0 | 0 | 0 | 0 | | 0 | | 0 | | 0 | | 0 | | 0 | | 0 |
| H145 | 1 |  | | | | 2 | | | | F | 38 | 0 | 0 | 1 | 0 | 0 | 1 | 0 | 0 | 0 | 0 | | 0 | | 0 | | 0 | | 0 | | 0 | | 0 |
| H146 | 0 |  | | | | | | 0 | | F | 29 | 0 | 1 | 1 | 0 | 1 | 0 | 0 | 0 | 0 | 0 | | 0 | | 0 | | 0 | | 0 | | 0 | | 0 |
| H147 | 1 |  | | | | | | 2 | | F | 26 | 0 | 0 | 1 | 0 | 0 | 1 | 0 | 0 | 1 | 1 | | 1 | | 0 | | 0 | | 0 | | 0 | | 0 |
| H148 | 1 |  | | | | | | 2 | | F | 27 | 0 | 0 | 1 | 0 | 0 | 1 | 0 | 0 | 1 | 1 | | 1 | | 0 | | 0 | | 0 | | 0 | | 0 |
| H149 | 0 |  | | | | | | 0 | | F | 43 | 0 | 1 | 1 | 0 | 0 | 1 | 0 | 1 | 0 | 0 | | 0 | | 0 | | 0 | | 0 | | 0 | | 0 |
| H150 | 0 |  | | | | | | 0 | | F | 25 | 0 | 1 | 1 | 0 | 1 | 1 | 0 | 1 | 0 | 0 | | 0 | | 0 | | 0 | | 0 | | 0 | | 0 |
| H151 | 0 |  | | | | | | 0 | | M | 55 | 0 | 1 | 1 | 1 | 1 | 0 | 0 | 0 | 0 | 0 | | 0 | | 0 | | 0 | | 1 | | 0 | | 0 |
| H152 | 1 |  | | | | | | 1 | | F | 23 | 0 | 0 | 1 | 1 | 0 | 0 | 0 | 0 | 0 | 0 | | 0 | | 0 | | 0 | | 0 | | 0 | | 0 |
| H153 | 1 |  | | | | | | 3 | | M | 14 | 0 | 1 | 1 | 1 | 0 | 1 | 0 | 0 | 0 | 0 | | 0 | | 0 | | 0 | | 0 | | 0 | | 0 |
| H154 | 0 |  | | | | | | 0 | | M | 23 | 0 | 1 | 0 | 1 | 0 | 1 | 0 | 0 | 0 | 0 | | 0 | | 0 | | 0 | | 0 | | 0 | | 0 |
| H155 | 0 |  | | | | | | 0 | | F | 21 | 0 | 1 | 0 | 0 | 0 | 0 | 1 | 0 | 0 | 0 | | 0 | | 0 | | 0 | | 0 | | 0 | | 0 |
| H156 | 0 |  | | | | | | 0 | | M | 14 | 0 | 1 | 1 | 1 | 1 | 1 | 0 | 0 | 1 | 1 | | 1 | | 0 | | 0 | | 1 | | 0 | | 0 |
| H157 | 0 |  | | | | | | 0 | | M | 34 | 0 | 1 | 1 | 1 | 1 | 1 | 0 | 0 | 0 | 0 | | 0 | | 0 | | 0 | | 0 | | 0 | | 0 |
| H158 | 1 |  | | | | | | 1 | | M | 23 | 0 | 0 | 1 | 1 | 0 | 0 | 0 | 0 | 0 | 0 | | 0 | | 0 | | 0 | | 0 | | 0 | | 0 |
| H159 | 1 |  | | | | | | 1 | | M | 30 | 0 | 1 | 1 | 1 | 0 | 0 | 0 | 0 | 0 | 0 | | 0 | | 0 | | 0 | | 0 | | 0 | | 0 |
| H160 | 0 |  | | | | | | 0 | | M | 40 | 0 | 1 | 1 | 1 | 1 | 0 | 0 | 0 | 0 | 0 | | 0 | | 0 | | 0 | | 0 | | 0 | | 0 |
| H161 | 1 |  | | | | | | 2 | | M | 25 | 0 | 1 | 0 | 0 | 0 | 1 | 0 | 0 | 0 | 0 | | 0 | | 0 | | 0 | | 0 | | 0 | | 0 |
| H162 | 1 |  | | | | | | 1 | | M | 16 | 0 | 1 | 1 | 1 | 0 | 1 | 0 | 0 | 0 | 0 | | 0 | | 0 | | 0 | | 0 | | 0 | | 0 |
| H163 | 0 |  | | | | | | 0 | | M | 29 | 0 | 1 | 1 | 1 | 1 | 1 | 0 | 0 | 0 | 0 | | 0 | | 0 | | 0 | | 0 | | 0 | | 0 |
| H164 | 1 |  | | | | | | 3 | | M | 25 | 0 | 1 | 1 | 1 | 0 | 1 | 0 | 0 | 0 | 0 | | 0 | | 0 | | 0 | | 0 | | 0 | | 0 |
| H165 | 1 |  | | | | | | 3 | | M | 28 | 0 | 0 | 1 | 0 | 0 | 1 | 0 | 0 | 0 | 0 | | 0 | | 0 | | 0 | | 0 | | 0 | | 0 |
| H166 | 0 |  | | | | | | 0 | | F | 15 | 0 | 1 | 1 | 0 | 1 | 0 | 0 | 0 | 0 | 0 | | 0 | | 0 | | 0 | | 0 | | 0 | | 0 |
| H167 | 1 |  | | | | | | 1 | | M | 35 | 0 | 0 | 1 | 0 | 0 | 1 | 0 | 0 | 0 | 0 | | 0 | | 0 | | 0 | | 0 | | 0 | | 0 |
| H168 | 0 |  | | | | | | 0 | | M | 30 | 0 | 1 | 1 | 1 | 1 | 1 | 0 | 0 | 0 | 0 | | 0 | | 0 | | 0 | | 0 | | 0 | | 0 |
| H169 | 1 |  | | | | | | 1 | | F | 25 | 0 | 0 | 1 | 1 | 0 | 1 | 0 | 0 | 0 | 0 | | 0 | | 0 | | 0 | | 0 | | 0 | | 0 |
| H170 | 0 |  | | | | | | 0 | | M | 50 | 0 | 1 | 1 | 0 | 1 | 1 | 0 | 0 | 0 | 0 | | 0 | | 0 | | 0 | | 0 | | 0 | | 0 |
| H171 | 1 |  | | | | | | 1 | | F | 52 | 0 | 0 | 1 | 1 | 1 | 1 | 0 | 0 | 0 | 0 | | 0 | | 0 | | 0 | | 0 | | 0 | | 0 |
| H172 | 0 |  | | 0 | | | | | | M | 17 | 0 | 1 | 1 | 1 | 0 | 1 | 0 | 0 | 0 | 0 | | 0 | | 0 | | 0 | | 0 | | 0 | | 0 |
| H173 | 1 |  | | 1 | | | | | | F | 28 | 0 | 0 | 1 | 1 | 0 | 1 | 0 | 0 | 0 | 0 | | 0 | | 0 | | 0 | | 0 | | 0 | | 0 |
| H174 | 1 |  | | 2 | | | | | | F | 18 | 0 | 0 | 1 | 0 | 0 | 1 | 0 | 0 | 0 | 1 | | 1 | | 0 | | 0 | | 1 | | 0 | | 0 |
| H175 | 1 |  | | 2 | | | | | | F | 16 | 0 | 0 | 1 | 1 | 0 | 1 | 0 | 0 | 0 | 0 | | 0 | | 0 | | 0 | | 0 | | 0 | | 0 |
| H176 | 0 |  | | 0 | | | | | | F | 25 | 0 | 1 | 1 | 1 | 0 | 1 | 0 | 0 | 0 | 0 | | 0 | | 0 | | 0 | | 0 | | 0 | | 0 |
| H177 | 1 |  | | 1 | | | | | | M | 30 | 0 | 0 | 0 | 0 | 0 | 1 | 0 | 0 | 0 | 0 | | 0 | | 0 | | 0 | | 0 | | 0 | | 0 |
| H178 | 1 |  | | 3 | | | | | | F | 20 | 0 | 0 | 1 | 0 | 0 | 1 | 0 | 1 | 0 | 0 | | 0 | | 0 | | 0 | | 0 | | 0 | | 0 |
| H179 | 1 |  | | 1 | | | | | | F | 27 | 0 | 0 | 1 | 0 | 0 | 1 | 0 | 0 | 0 | 0 | | 0 | | 0 | | 0 | | 0 | | 0 | | 0 |
| H180 | 0 |  | | 0 | | | | | | F | 50 | 0 | 1 | 1 | 1 | 0 | 1 | 0 | 1 | 0 | 0 | | 0 | | 0 | | 0 | | 0 | | 0 | | 0 |
| H181 | 0 |  | | 0 | | | | | | M | 15 | 0 | 1 | 1 | 1 | 0 | 1 | 0 | 0 | 0 | 0 | | 0 | | 0 | | 0 | | 0 | | 0 | | 0 |
| H182 | 1 |  | | 3 | | | | | | M | 25 | 0 | 1 | 1 | 1 | 0 | 1 | 0 | 0 | 1 | 1 | | 1 | | 0 | | 0 | | 0 | | 0 | | 0 |
| H183 | 1 |  | | 1 | | | | | | F | 37 | 0 | 0 | 1 | 1 | 1 | 1 | 0 | 0 | 0 | 0 | | 0 | | 0 | | 0 | | 0 | | 0 | | 0 |
| H184 | 1 |  | | 1 | | | | | | F | 18 | 0 | 1 | 1 | 0 | 0 | 0 | 0 | 0 | 0 | 0 | | 0 | | 0 | | 0 | | 0 | | 0 | | 0 |
| H185 | 0 |  | | 0 | | | | | | F | 14 | 0 | 1 | 1 | 0 | 1 | 1 | 0 | 0 | 0 | 0 | | 0 | | 0 | | 0 | | 0 | | 0 | | 0 |
| H186 | 1 |  | | 2 | | | | | | M | 35 | 0 | 0 | 1 | 1 | 0 | 1 | 0 | 0 | 0 | 0 | | 0 | | 0 | | 0 | | 0 | | 0 | | 0 |
| H187 | 0 |  | | 0 | | | | | | M | 34 | 1 | 1 | 1 | 1 | 1 | 0 | 0 | 0 | 0 | 0 | | 0 | | 0 | | 0 | | 1 | | 0 | | 0 |
| H188 | 0 |  | | 0 | | | | | | M | 50 | 0 | 1 | 1 | 1 | 1 | 1 | 0 | 1 | 0 | 0 | | 0 | | 0 | | 0 | | 1 | | 0 | | 0 |
| H189 | 1 |  | | 2 | | | | | | F | 41 | 0 | 0 | 1 | 0 | 0 | 1 | 0 | 0 | 0 | 0 | | 0 | | 0 | | 0 | | 0 | | 0 | | 0 |
| H190 | 1 |  | | 2 | | | | | | F | 27 | 0 | 0 | 1 | 0 | 0 | 0 | 0 | 1 | 0 | 0 | | 0 | | 0 | | 0 | | 0 | | 0 | | 0 |
| H191 | 1 |  | | 1 | | | | | | M | 40 | 0 | 0 | 1 | 0 | 0 | 1 | 0 | 0 | 0 | 0 | | 0 | | 0 | | 0 | | 0 | | 0 | | 0 |
| H192 | 1 |  | | 1 | | | | | | F | 25 | 0 | 0 | 1 | 1 | 0 | 1 | 0 | 0 | 1 | 0 | | 0 | | 0 | | 0 | | 0 | | 0 | | 0 |
| H193 | 0 |  | | 0 | | | | | | M | 30 | 0 | 1 | 1 | 1 | 1 | 0 | 0 | 0 | 0 | 0 | | 0 | | 0 | | 0 | | 0 | | 0 | | 0 |
| H194 | 1 |  | | 3 | | | | | | M | 25 | 0 | 1 | 1 | 1 | 0 | 0 | 0 | 0 | 0 | 0 | | 0 | | 0 | | 0 | | 0 | | 0 | | 0 |
| H195 | 0 |  | | 0 | | | | | | F | 20 | 0 | 1 | 1 | 1 | 1 | 0 | 0 | 1 | 0 | 0 | | 0 | | 0 | | 0 | | 0 | | 0 | | 0 |
| H196 | 0 |  | | 0 | | | | | | M | 14 | 0 | 1 | 1 | 1 | 1 | 1 | 1 | 0 | 0 | 0 | | 0 | | 0 | | 0 | | 0 | | 0 | | 0 |
| H197 | 1 |  | | 1 | | | | | | F | 14 | 0 | 0 | 1 | 0 | 0 | 1 | 0 | 0 | 0 | 0 | | 0 | | 0 | | 0 | | 0 | | 0 | | 0 |
| H198 | 1 |  | | | | | | 1 | | M | 30 | 0 | 1 | 1 | 0 | 0 | 0 | 0 | 0 | 0 | 0 | | 0 | | 0 | | 0 | | 0 | | 0 | | 0 |
| H199 | 0 |  | | | | | | 0 | | F | 30 | 1 | 1 | 1 | 1 | 0 | 1 | 0 | 0 | 0 | 0 | | 0 | | 0 | | 0 | | 0 | | 0 | | 0 |
| H200 | 1 |  | | | | | | 1 | | M | 30 | 0 | 1 | 1 | 0 | 0 | 0 | 0 | 0 | 0 | 0 | | 0 | | 0 | | 0 | | 0 | | 0 | | 0 |
| H201 | 0 |  | | | | | | 0 | | M | 18 | 0 | 1 | 1 | 1 | 0 | 1 | 0 | 0 | 0 | 0 | | 0 | | 0 | | 0 | | 0 | | 0 | | 0 |
| H202 | 1 |  | | | | | | 1 | | M | 14 | 0 | 1 | 0 | 0 | 0 | 1 | 0 | 0 | 0 | 0 | | 0 | | 0 | | 0 | | 0 | | 0 | | 0 |
| H203 | 0 |  | | | | | | 0 | | F | 27 | 0 | 1 | 1 | 1 | 0 | 0 | 1 | 0 | 0 | 0 | | 0 | | 0 | | 0 | | 0 | | 0 | | 0 |
| H204 | 1 |  | | | | | | 1 | | M | 25 | 0 | 1 | 1 | 0 | 0 | 1 | 0 | 0 | 0 | 0 | | 0 | | 0 | | 0 | | 0 | | 0 | | 0 |
| H205 | 1 |  | | | | | | 2 | | M | 44 | 0 | 0 | 1 | 0 | 0 | 1 | 0 | 0 | 0 | 0 | | 0 | | 0 | | 0 | | 0 | | 0 | | 0 |
| H206 | 1 |  | | | | | | 2 | | M | 23 | 0 | 0 | 1 | 1 | 0 | 1 | 0 | 0 | 0 | 0 | | 0 | | 0 | | 0 | | 0 | | 0 | | 0 |
| H207 | 1 |  | | | | | | 2 | | M | 19 | 0 | 1 | 1 | 0 | 0 | 1 | 0 | 0 | 0 | 0 | | 0 | | 0 | | 0 | | 0 | | 0 | | 0 |
| H208 | 1 |  | | | | | | 2 | | F | 20 | 0 | 1 | 1 | 0 | 0 | 0 | 0 | 0 | 0 | 0 | | 0 | | 0 | | 0 | | 0 | | 0 | | 0 |
| H209 | 1 |  | | | | | | 1 | | M | 22 | 0 | 1 | 1 | 1 | 0 | 1 | 0 | 0 | 1 | 1 | | 1 | | 0 | | 0 | | 0 | | 0 | | 0 |
| H210 | 1 |  | | | | | | 1 | | F | 30 | 0 | 1 | 1 | 0 | 0 | 1 | 0 | 0 | 0 | 0 | | 1 | | 0 | | 0 | | 0 | | 0 | | 0 |
| H211 | 0 |  | | | | | | 0 | | F | 30 | 0 | 1 | 1 | 1 | 1 | 1 | 0 | 1 | 0 | 0 | | 0 | | 0 | | 0 | | 0 | | 0 | | 0 |
| H212 | 0 |  | | | | | | 0 | | M | 20 | 0 | 1 | 1 | 1 | 1 | 0 | 0 | 0 | 0 | 0 | | 0 | | 0 | | 0 | | 1 | | 0 | | 0 |
| H213 | 1 |  | | | | | | 1 | | F | 25 | 0 | 0 | 0 | 0 | 0 | 1 | 0 | 0 | 0 | 0 | | 0 | | 0 | | 0 | | 0 | | 0 | | 0 |
| H214 | 1 |  | | | | | | 1 | | F | 60 | 0 | 0 | 1 | 0 | 0 | 0 | 0 | 0 | 0 | 0 | | 0 | | 0 | | 0 | | 1 | | 0 | | 0 |
| H215 | 0 |  | | | | | | 0 | | F | 20 | 0 | 1 | 1 | 0 | 1 | 1 | 0 | 0 | 0 | 0 | | 0 | | 0 | | 0 | | 0 | | 0 | | 0 |
| H216 | 1 |  | | | | | | 2 | | F | 35 | 0 | 1 | 1 | 1 | 0 | 1 | 0 | 0 | 0 | 0 | | 0 | | 0 | | 0 | | 0 | | 0 | | 0 |
| H217 | 1 |  | | | | | | 1 | | M | 20 | 0 | 1 | 1 | 0 | 0 | 0 | 0 | 0 | 0 | 0 | | 0 | | 0 | | 0 | | 0 | | 0 | | 0 |
| H218 | 1 |  | | | | | | 1 | | M | 20 | 0 | 0 | 1 | 1 | 0 | 0 | 0 | 0 | 0 | 0 | | 0 | | 0 | | 0 | | 0 | | 0 | | 0 |
| H219 | 1 |  | | | | | | 1 | | F | 38 | 0 | 0 | 1 | 1 | 0 | 0 | 0 | 0 | 0 | 1 | | 1 | | 0 | | 0 | | 0 | | 0 | | 0 |
| H220 | 1 |  | | | | | | 1 | | F |  |  | 0 | 1 | 1 | 0 | 1 | 0 | 0 | 0 | 0 | | 0 | | 0 | | 0 | | 0 | | 0 | | 0 |
| H221 | 1 |  | | | | | | 1 | | M | 27 | 0 | 0 | 1 | 1 | 0 | 1 | 0 | 0 | 0 | 0 | | 0 | | 0 | | 0 | | 0 | | 0 | | 0 |
| H222 | 1 |  | | | | | | 3 | | F | 40 | 0 | 1 | 1 | 1 | 0 | 1 | 0 | 0 | 0 | 0 | | 0 | | 0 | | 0 | | 0 | | 0 | | 0 |
| H223 | 0 |  | | | | | | 0 | | F | 25 | 0 | 1 | 1 | 1 | 0 | 1 | 0 | 0 | 0 | 0 | | 0 | | 0 | | 0 | | 0 | | 0 | | 0 |
| H224 | 0 |  | | | 0 | | | | | M | 55 | 0 | 1 | 1 | 0 | 0 | 1 | 0 | 1 | 0 | 0 | | 0 | | 0 | | 0 | | 1 | | 0 | | 0 |
| H225 | 0 |  | | | 0 | | | | | F | 23 | 0 | 1 | 1 | 0 | 1 | 1 | 0 | 1 | 0 | 1 | | 1 | | 0 | | 0 | | 0 | | 0 | | 0 |
| H226 | 0 |  | | | 0 | | | | | M | 14 | 0 | 1 | 1 | 0 | 1 | 0 | 0 | 0 | 0 | 0 | | 0 | | 0 | | 0 | | 1 | | 0 | | 0 |
| H227 | 1 |  | | | 1 | | | | | F | 23 | 0 | 0 | 1 | 0 | 0 | 1 | 0 | 0 | 0 | 0 | | 0 | | 0 | | 0 | | 0 | | 0 | | 0 |
| H228 | 0 |  | | | 0 | | | | | F | 21 | 0 | 1 | 1 | 1 | 1 | 0 | 0 | 0 | 0 | 0 | | 0 | | 0 | | 0 | | 1 | | 0 | | 0 |
| H229 | 0 |  | | | 0 | | | | | M | 14 | 0 | 1 | 1 | 1 | 1 | 1 | 0 | 0 | 0 | 0 | | 0 | | 0 | | 0 | | 0 | | 0 | | 0 |
| H230 | 1 |  | | | 1 | | | | | M | 34 | 0 | 1 | 1 | 0 | 0 | 1 | 0 | 0 | 0 | 0 | | 0 | | 0 | | 0 | | 0 | | 0 | | 0 |
| H231 | 0 |  | | | 0 | | | | | M | 23 | 0 | 1 | 1 | 1 | 0 | 0 | 0 | 0 | 0 | 0 | | 0 | | 0 | | 0 | | 0 | | 0 | | 0 |
| H232 | 0 |  | | | 0 | | | | | M | 30 | 0 | 1 | 1 | 1 | 0 | 1 | 0 | 0 | 0 | 0 | | 0 | | 0 | | 0 | | 0 | | 0 | | 0 |
| H233 | 1 |  | | | 1 | | | | | M | 40 | 0 | 1 | 1 | 0 | 0 | 1 | 0 | 0 | 1 | 0 | | 0 | | 0 | | 0 | | 0 | | 0 | | 0 |
| H234 | 0 |  | | | 0 | | | | | M | 25 | 0 | 1 | 1 | 1 | 1 | 1 | 0 | 0 | 0 | 0 | | 0 | | 0 | | 0 | | 0 | | 0 | | 0 |
| H235 | 0 |  | | | 0 | | | | | F | 16 | 0 | 1 | 1 | 1 | 1 | 0 | 0 | 1 | 0 | 0 | | 0 | | 0 | | 0 | | 0 | | 0 | | 0 |
| H236 | 1 |  | | | 1 | | | | | M | 29 | 0 | 1 | 1 | 0 | 0 | 1 | 0 | 0 | 0 | 0 | | 0 | | 0 | | 0 | | 0 | | 0 | | 0 |
| H237 | 1 |  | | | 1 | | | | | M | 25 | 0 | 0 | 1 | 0 | 0 | 1 | 0 | 0 | 1 | 0 | | 0 | | 0 | | 0 | | 0 | | 0 | | 0 |
| H238 | 1 |  | | | 1 | | | | | M | 28 | 0 | 0 | 1 | 0 | 0 | 0 | 0 | 0 | 0 | 0 | | 0 | | 0 | | 0 | | 0 | | 0 | | 0 |
| H239 | 1 |  | | | 3 | | | | | F | 15 | 0 | 1 | 1 | 0 | 0 | 1 | 0 | 1 | 1 | 0 | | 0 | | 0 | | 0 | | 0 | | 0 | | 0 |
| H240 | 1 |  | | | 1 | | | | | M | 35 | 0 | 1 | 1 | 1 | 0 | 1 | 0 | 0 | 0 | 0 | | 0 | | 0 | | 0 | | 0 | | 0 | | 0 |
| H241 | 1 |  | | | 3 | | | | | M | 30 | 0 | 0 | 1 | 1 | 0 | 0 | 0 | 0 | 0 | 0 | | 0 | | 0 | | 0 | | 0 | | 0 | | 0 |
| H242 | 1 |  | | | 1 | | | | | F | 25 | 0 | 0 | 1 | 1 | 0 | 0 | 0 | 1 | 0 | 0 | | 0 | | 0 | | 0 | | 0 | | 0 | | 0 |
| H243 | 1 |  | | | 1 | | | | | M | 50 | 0 | 1 | 1 | 1 | 0 | 0 | 0 | 0 | 1 | 0 | | 0 | | 0 | | 0 | | 0 | | 0 | | 0 |
| H244 | 0 |  | | | 0 | | | | | F | 52 | 1 | 1 | 1 | 0 | 1 | 0 | 0 | 1 | 0 | 0 | | 0 | | 0 | | 0 | | 0 | | 0 | | 0 |
| H245 | 0 |  | | | 0 | | | | | M | 17 | 0 | 1 | 1 | 1 | 1 | 0 | 0 | 0 | 1 | 0 | | 0 | | 0 | | 0 | | 0 | | 0 | | 0 |
| H246 | 0 |  | | | 0 | | | | | F | 28 | 0 | 1 | 1 | 1 | 0 | 0 | 0 | 1 | 0 | 0 | | 0 | | 0 | | 0 | | 0 | | 0 | | 0 |
| H247 | 1 |  | | | 2 | | | | | F | 18 | 0 | 1 | 1 | 0 | 0 | 1 | 0 | 0 | 0 | 0 | | 0 | | 0 | | 0 | | 0 | | 0 | | 0 |
| H248 | 1 |  | | | 1 | | | | | F | 16 | 0 | 0 | 1 | 0 | 0 | 1 | 0 | 0 | 1 | 0 | | 0 | | 0 | | 0 | | 0 | | 0 | | 0 |
| H249 | 1 |  | | | 2 | | | | | F | 25 | 0 | 0 | 1 | 0 | 0 | 1 | 0 | 0 | 0 | 0 | | 0 | | 0 | | 0 | | 0 | | 0 | | 0 |
| H250 | 1 |  | | 1 | | | | | | M | 30 | 0 | 0 | 1 | 0 | 0 | 1 | 0 | 0 | 0 | 0 | | 0 | | 0 | | 0 | | 0 | | 0 | | 0 |
| H251 | 0 |  | | 0 | | | | | | F | 20 | 0 | 1 | 1 | 1 | 1 | 0 | 0 | 1 | 0 | 0 | | 0 | | 0 | | 0 | | 0 | | 0 | | 0 |
| H252 | 1 |  | | 1 | | | | | | F | 27 | 0 | 0 | 0 | 1 | 0 | 1 | 0 | 0 | 0 | 0 | | 0 | | 0 | | 0 | | 0 | | 0 | | 0 |
| H253 | 0 |  | | 0 | | | | | | F | 50 | 0 | 1 | 1 | 1 | 0 | 1 | 0 | 0 | 0 | 0 | | 0 | | 0 | | 0 | | 0 | | 0 | | 0 |
| H254 | 1 |  | | 2 | | | | | | M | 15 | 0 | 1 | 1 | 1 | 0 | 0 | 0 | 0 | 0 | 0 | | 0 | | 0 | | 0 | | 0 | | 0 | | 0 |
| H255 | 0 |  | | 0 | | | | | | M | 25 | 0 | 1 | 1 | 1 | 1 | 1 | 1 | 0 | 0 | 0 | | 0 | | 0 | | 0 | | 0 | | 0 | | 0 |
| H256 | 0 |  | | 0 | | | | | | F | 37 | 0 | 1 | 1 | 1 | 1 | 1 | 0 | 0 | 0 | 0 | | 0 | | 0 | | 0 | | 1 | | 0 | | 0 |
| H257 | 1 |  | | 1 | | | | | | M | 18 | 0 | 0 | 1 | 0 | 0 | 0 | 0 | 0 | 0 | 0 | | 0 | | 0 | | 0 | | 0 | | 0 | | 0 |
| H258 | 0 |  | | 0 | | | | | | F | 14 | 0 | 1 | 1 | 1 | 0 | 1 | 0 | 1 | 0 | 0 | | 0 | | 0 | | 0 | | 0 | | 0 | | 0 |
| H259 | 1 |  | | 1 | | | | | | M | 35 | 0 | 1 | 1 | 0 | 0 | 1 | 0 | 0 | 0 | 0 | | 0 | | 0 | | 0 | | 0 | | 0 | | 0 |
| H260 | 1 |  | | 2 | | | | | | M | 34 | 0 | 0 | 1 | 0 | 0 | 0 | 0 | 0 | 0 | 0 | | 0 | | 0 | | 0 | | 0 | | 0 | | 0 |
| H261 | 1 |  | | 1 | | | | | | M | 50 | 0 | 0 | 1 | 0 | 0 | 1 | 0 | 0 | 1 | 0 | | 0 | | 0 | | 0 | | 0 | | 0 | | 0 |
| H262 | 0 |  | | 0 | | | | | | F | 41 | 0 | 1 | 1 | 1 | 0 | 1 | 0 | 0 | 0 | 0 | | 0 | | 0 | | 0 | | 0 | | 0 | | 0 |
| H263 | 0 |  | | 0 | | | | | | F | 27 | 0 | 0 | 1 | 1 | 1 | 0 | 0 | 1 | 0 | 0 | | 0 | | 0 | | 0 | | 0 | | 0 | | 0 |
| H264 | 0 |  | | 0 | | | | | | M | 40 | 0 | 0 | 1 | 1 | 1 | 1 | 0 | 0 | 0 | 0 | | 0 | | 0 | | 0 | | 1 | | 0 | | 0 |
| H265 | 1 |  | | 2 | | | | | | M | 25 | 0 | 1 | 1 | 0 | 0 | 1 | 0 | 0 | 0 | 0 | | 0 | | 0 | | 0 | | 0 | | 0 | | 0 |
| H266 | 0 |  | | 0 | | | | | | M | 30 | 0 | 1 | 1 | 1 | 1 | 1 | 0 | 0 | 0 | 0 | | 0 | | 0 | | 0 | | 0 | | 0 | | 0 |
| J267 | 1 |  | | 1 | | | | | | M | 25 | 0 | 0 | 1 | 1 | 0 | 0 | 0 | 0 | 0 | 0 | | 0 | | 0 | | 0 | | 0 | | 0 | | 0 |
| J268 | 1 |  | | 3 | | | | | | F | 20 | 0 | 0 | 1 | 1 | 0 | 1 | 0 | 0 | 0 | 0 | | 0 | | 0 | | 0 | | 0 | | 0 | | 0 |
| J269 | 1 |  | | 1 | | | | | | M | 14 | 0 | 1 | 1 | 1 | 0 | 1 | 0 | 0 | 0 | 0 | | 0 | | 0 | | 0 | | 0 | | 0 | | 0 |
| J270 | 1 |  | | 1 | | | | | | F | 14 | 0 | 1 | 1 | 1 | 0 | 0 | 0 | 0 | 0 | 0 | | 0 | | 0 | | 0 | | 0 | | 0 | | 0 |
| J271 | 0 |  | | 0 | | | | | | M | 30 | 0 | 1 | 1 | 1 | 0 | 1 | 0 | 0 | 0 | 0 | | 0 | | 0 | | 0 | | 0 | | 0 | | 0 |
| J272 | 0 |  | | 0 | | | | | | F | 30 | 1 | 1 | 1 | 1 | 1 | 1 | 0 | 1 | 0 | 0 | | 0 | | 0 | | 0 | | 0 | | 0 | | 0 |
| J273 | 1 |  | | 3 | | | | | | F | 30 | 0 | 0 | 1 | 0 | 0 | 0 | 0 | 0 | 0 | 0 | | 0 | | 0 | | 0 | | 0 | | 0 | | 0 |
| J274 | 0 |  | | 0 | | | | | | M | 18 | 0 | 1 | 1 | 0 | 1 | 1 | 0 | 0 | 0 | 0 | | 0 | | 0 | | 0 | | 0 | | 0 | | 0 |
| J275 | 1 |  | | 3 | | | | | | M | 14 | 0 | 1 | 1 | 1 | 0 | 0 | 0 | 0 | 0 | 0 | | 0 | | 0 | | 0 | | 0 | | 0 | | 0 |
| J276 | 1 |  | | | 2 | | | | | F | 27 | 0 | 1 | 1 | 0 | 0 | 1 | 0 | 0 | 1 | 0 | | 0 | | 0 | | 0 | | 0 | | 0 | | 0 |
| J277 | 0 |  | | | 0 | | | | | M | 25 | 0 | 1 | 1 | 1 | 0 | 1 | 0 | 0 | 0 | 0 | | 0 | | 0 | | 0 | | 0 | | 0 | | 0 |
| J278 | 0 |  | | | 0 | | | | | M | 36 | 0 | 1 | 1 | 1 | 1 | 1 | 0 | 0 | 1 | 0 | | 0 | | 0 | | 0 | | 0 | | 0 | | 0 |
| J279 | 0 |  | | | 0 | | | | | M | 23 | 0 | 1 | 1 | 1 | 1 | 1 | 0 | 0 | 0 | 0 | | 0 | | 0 | | 0 | | 0 | | 0 | | 0 |
| J280 | 1 |  | | | 1 | | | | | M | 19 | 0 | 0 | 1 | 1 | 0 | 0 | 0 | 0 | 0 | 0 | | 0 | | 0 | | 0 | | 0 | | 0 | | 0 |
| J281 | 1 |  | | | 1 | | | | | F | 20 | 0 | 1 | 1 | 0 | 0 | 1 | 0 | 0 | 1 | 0 | | 0 | | 0 | | 0 | | 0 | | 0 | | 0 |
| J282 | 0 |  | | | 0 | | | | | M | 22 | 0 | 1 | 1 | 0 | 1 | 1 | 0 | 0 | 0 | 0 | | 0 | | 0 | | 0 | | 0 | | 0 | | 0 |
| J283 | 1 |  | | | 1 | | | | | F | 30 | 0 | 0 | 1 | 0 | 0 | 0 | 0 | 0 | 0 | 0 | | 0 | | 0 | | 0 | | 0 | | 0 | | 0 |
| J284 | 1 |  | | | 1 | | | | | F | 30 | 0 | 0 | 1 | 0 | 0 | 0 | 0 | 0 | 1 | 0 | | 0 | | 0 | | 0 | | 0 | | 0 | | 0 |
| J285 | 0 |  | | | 1 | | | | | f | 20 | 0 | 1 | 1 | 1 | 0 | 1 | 1 | 1 | 0 | 1 | | 1 | | 0 | | 0 | | 0 | | 0 | | 0 |
| J286 | 1 |  | | | 1 | | | | | M | 25 | 0 | 0 | 1 | 1 | 0 | 0 | 0 | 0 | 0 | 0 | | 0 | | 0 | | 0 | | 0 | | 0 | | 0 |
| J287 | 1 |  | | | 2 | | | | | M | 60 | 0 | 1 | 1 | 0 | 0 | 1 | 0 | 1 | 0 | 0 | | 0 | | 0 | | 0 | | 0 | | 0 | | 0 |
| J288 | 1 |  | | | 1 | | | | | F | 20 | 0 | 1 | 1 | 0 | 0 | 1 | 0 | 0 | 0 | 0 | | 0 | | 0 | | 0 | | 0 | | 0 | | 0 |
| J289 | 1 |  | | | 3 | | | | | F | 35 | 0 | 1 | 1 | 1 | 0 | 0 | 0 | 1 | 0 | 0 | | 0 | | 0 | | 0 | | 0 | | 0 | | 0 |
| J290 | 1 |  | | | 1 | | | | | M | 20 | 0 | 1 | 1 | 0 | 0 | 1 | 0 | 0 | 1 | 0 | | 0 | | 0 | | 0 | | 0 | | 0 | | 0 |
| J291 | 1 |  | | | 1 | | | | | F | 20 | 0 | 1 | 1 | 1 | 0 | 1 | 0 | 1 | 0 | 0 | | 0 | | 0 | | 0 | | 0 | | 0 | | 0 |
| J292 | 0 |  | | | 0 | | | | | M | 38 | 0 | 1 | 1 | 0 | 1 | 0 | 0 | 0 | 0 | 0 | | 0 | | 0 | | 0 | | 0 | | 0 | | 0 |
| J293 | 0 |  | | | 0 | | | | | M |  | 0 | 1 | 1 | 1 | 1 | 1 | 0 | 0 | 0 | 0 | | 0 | | 0 | | 0 | | 0 | | 0 | | 0 |
| J294 | 1 |  | | | 2 | | | | | M | 27 | 0 | 1 | 0 | 1 | 0 | 0 | 0 | 0 | 0 | 0 | | 0 | | 0 | | 0 | | 0 | | 0 | | 0 |
| J295 | 0 |  | | | 0 | | | | | F | 40 | 0 | 1 | 1 | 1 | 0 | 1 | 0 | 1 | 0 | 0 | | 0 | | 0 | | 0 | | 0 | | 0 | | 0 |
| J296 | 1 |  | | | 3 | | | | | M | 25 | 0 | 0 | 1 | 0 | 1 | 1 | 0 | 0 | 0 | 0 | | 0 | | 0 | | 0 | | 0 | | 0 | | 0 |
| J297 | 0 |  | | | 0 | | | | | M | 55 | 0 | 1 | 1 | 1 | 1 | 1 | 0 | 0 | 0 | 0 | | 0 | | 0 | | 0 | | 0 | | 0 | | 0 |
| J298 | 1 |  | | | 1 | | | | | F | 23 | 0 | 1 | 1 | 1 | 1 | 0 | 0 | 0 | 0 | 0 | | 0 | | 0 | | 0 | | 0 | | 0 | | 0 |
| J299 | 0 |  | | | 0 | | | | | F | 14 | 0 | 1 | 1 | 1 | 0 | 1 | 0 | 1 | 0 | 0 | | 0 | | 0 | | 0 | | 1 | | 0 | | 0 |
| J300 | 0 |  | | | 0 | | | | | M | 23 | 0 | 1 | 1 | 1 | 0 | 0 | 0 | 1 | 1 | 0 | | 0 | | 0 | | 0 | | 0 | | 0 | | 0 |
| J301 | 1 |  | | | 1 | | | | | F | 21 | 0 | 1 | 1 | 1 | 0 | 1 | 0 | 0 | 0 | 0 | | 0 | | 0 | | 0 | | 0 | | 0 | | 0 |
| J302 | 0 |  | | | 0 | | | | | M | 14 | 0 | 1 | 1 | 1 | 0 | 1 | 0 | 1 | 0 | 0 | | 0 | | 0 | | 0 | | 0 | | 0 | | 0 |
| J303 | 1 |  | | | 1 | | | | | F | 34 | 0 | 0 | 1 | 0 | 0 | 0 | 0 | 1 | 0 | 0 | | 0 | | 0 | | 0 | | 0 | | 0 | | 0 |
| J304 | 1 |  | | | 1 | | | | | M | 23 | 0 | 1 | 1 | 0 | 1 | 1 | 0 | 0 | 0 | 0 | | 0 | | 0 | | 0 | | 0 | | 0 | | 0 |
| J305 | 0 |  | | | 0 | | | | | F | 30 | 0 | 0 | 1 | 1 | 1 | 1 | 0 | 1 | 0 | 0 | | 0 | | 0 | | 0 | | 0 | | 0 | | 0 |
| J306 | 1 |  | | | 1 | | | | | M | 40 | 0 | 1 | 1 | 0 | 0 | 0 | 0 | 0 | 0 | 0 | | 0 | | 0 | | 0 | | 0 | | 0 | | 0 |
| J307 | 1 |  | | | 2 | | | | | M | 25 | 0 | 1 | 1 | 1 | 1 | 1 | 0 | 0 | 1 | 0 | | 0 | | 0 | | 0 | | 0 | | 0 | | 0 |
| J308 | 0 |  | | | 0 | | | | | F | 16 | 0 | 1 | 1 | 1 | 1 | 0 | 0 | 0 | 0 | 0 | | 0 | | 0 | | 0 | | 0 | | 0 | | 0 |
| J309 | 1 |  | | | 1 | | | | | M | 29 | 0 | 1 | 1 | 1 | 0 | 1 | 0 | 1 | 0 | 0 | | 0 | | 0 | | 0 | | 0 | | 0 | | 0 |
| J310 | 1 |  | | | 1 | | | | | M | 25 | 0 | 1 | 1 | 1 | 0 | 1 | 0 | 0 | 0 | 0 | | 0 | | 0 | | 0 | | 0 | | 0 | | 0 |
| J311 | 1 |  | | | 1 | | | | | M | 28 | 0 | 0 | 1 | 0 | 0 | 0 | 0 | 1 | 0 | 0 | | 0 | | 0 | | 0 | | 0 | | 0 | | 0 |
| J312 | 1 |  | | | 2 | | | | | F | 15 | 0 | 1 | 1 | 0 | 0 | 1 | 0 | 0 | 1 | 0 | | 0 | | 0 | | 0 | | 0 | | 0 | | 0 |
| J313 | 0 |  | | | 0 | | | | | M | 35 | 0 | 1 | 1 | 0 | 0 | 1 | 0 | 0 | 0 | 0 | | 0 | | 0 | | 0 | | 1 | | 0 | | 0 |
| J314 | 0 |  | | | 0 | | | | | M | 30 | 0 | 1 | 1 | 1 | 0 | 0 | 0 | 0 | 1 | 0 | | 0 | | 0 | | 0 | | 1 | | 0 | | 0 |
| J315 | 0 |  | | | 0 | | | | | F | 25 | 1 | 1 | 1 | 1 | 1 | 1 | 0 | 1 | 0 | 0 | | 0 | | 0 | | 0 | | 1 | | 0 | | 0 |
| J316 | 0 |  | | | 0 | | | | | M | 50 | 0 | 1 | 1 | 1 | 1 | 1 | 0 | 0 | 0 | 0 | | 0 | | 0 | | 0 | | 0 | | 0 | | 0 |
| J317 | 0 |  | | | 0 | | | | | F | 52 | 0 | 1 | 1 | 1 | 0 | 0 | 0 | 1 | 0 | 0 | | 0 | | 0 | | 0 | | 0 | | 0 | | 0 |
| J318 | 1 |  | | | 1 | | | | | M | 19 | 0 | 1 | 1 | 0 | 0 | 1 | 0 | 0 | 0 | 0 | | 0 | | 0 | | 0 | | 0 | | 0 | | 0 |
| J319 | 1 |  | | | 1 | | | | | F | 32 | 0 | 1 | 1 | 0 | 0 | 0 | 0 | 0 | 0 | 0 | | 0 | | 0 | | 0 | | 0 | | 0 | | 0 |
| J320 | 1 |  | | | 3 | | | | | F | 23 | 0 | 1 | 1 | 0 | 0 | 0 | 0 | 0 | 0 | 0 | | 0 | | 0 | | 0 | | 0 | | 0 | | 0 |
| J321 | 1 |  | | | 1 | | | | | F | 18 | 0 | 1 | 1 | 0 | 0 | 1 | 0 | 1 | 1 | 0 | | 0 | | 0 | | 0 | | 0 | | 0 | | 0 |
| J322 | 1 |  | | | 1 | | | | | F | 38 | 0 | 1 | 1 | 0 | 0 | 1 | 0 | 0 | 0 | 0 | | 0 | | 0 | | 0 | | 0 | | 0 | | 0 |
| J323 | 1 |  | | | 2 | | | | | M | 35 | 0 | 1 | 1 | 0 | 0 | 0 | 0 | 0 | 0 | 0 | | 0 | | 0 | | 0 | | 0 | | 0 | | 0 |
| J324 | 0 |  | | | 0 | | | | | F | 21 | 0 | 1 | 1 | 0 | 0 | 0 | 0 | 1 | 0 | 0 | | 0 | | 0 | | 0 | | 0 | | 0 | | 0 |
| J325 | 1 |  | | | 2 | | | | | F | 30 | 0 | 1 | 1 | 0 | 0 | 0 | 0 | 0 | 0 | 0 | | 1 | | 0 | | 0 | | 0 | | 0 | | 0 |
| J326 | 1 |  | | | 1 | | | | | F | 18 | 0 | 0 | 1 | 0 | 0 | 0 | 0 | 0 | 0 | 0 | | 0 | | 0 | | 0 | | 0 | | 0 | | 0 |
| J327 | 1 |  | | | 1 | | | | | M | 17 | 0 | 1 | 1 | 1 | 0 | 1 | 0 | 0 | 1 | 0 | | 0 | | 0 | | 0 | | 0 | | 0 | | 0 |
| J328 | 0 |  | | | 0 | | | | | M | 15 | 0 | 1 | 1 | 1 | 0 | 1 | 0 | 0 | 0 | 0 | | 0 | | 0 | | 0 | | 1 | | 0 | | 0 |
| J329 | 0 |  | | | 0 | | | | | F | 18 | 0 | 1 | 1 | 0 | 0 | 1 | 0 | 1 | 0 | 0 | | 1 | | 0 | | 0 | | 0 | | 0 | | 0 |
| J330 | 0 |  | | | 0 | | | | | F | 38 | 0 | 1 | 1 | 1 | 1 | 1 | 0 | 1 | 0 | 0 | | 0 | | 0 | | 0 | | 1 | | 0 | | 0 |
| J331 | 1 |  | | | 1 | | | | | F | 23 | 0 | 1 | 1 | 1 | 0 | 0 | 0 | 0 | 0 | 0 | | 0 | | 0 | | 0 | | 0 | | 0 | | 0 |
| J332 | 0 |  | | | 0 | | | | | M | 39 | 0 | 1 | 1 | 1 | 1 | 1 | 0 | 0 | 0 | 0 | | 0 | | 0 | | 0 | | 0 | | 0 | | 0 |
| J333 | 1 |  | | | 3 | | | | | M | 49 | 0 | 0 | 1 | 0 | 0 | 1 | 0 | 1 | 0 | 0 | | 0 | | 0 | | 0 | | 0 | | 0 | | 0 |
| J334 | 0 |  | | | 0 | | | | | M | 45 | 0 | 1 | 1 | 1 | 0 | 1 | 0 | 0 | 0 | 0 | | 0 | | 0 | | 0 | | 0 | | 0 | | 0 |
| J335 | 1 |  | | | 1 | | | | | F | 30 | 0 | 1 | 1 | 1 | 0 | 0 | 0 | 0 | 0 | 0 | | 0 | | 0 | | 0 | | 0 | | 0 | | 0 |
| J336 | 1 |  | | | 2 | | | | | F | 38 | 0 | 1 | 1 | 0 | 0 | 1 | 0 | 1 | 0 | 0 | | 0 | | 0 | | 0 | | 0 | | 0 | | 0 |
| J337 | 1 |  | | | 1 | | | | | M | 39 | 0 | 1 | 1 | 0 | 0 | 0 | 0 | 0 | 0 | 0 | | 0 | | 0 | | 0 | | 0 | | 0 | | 0 |
| J338 | 1 |  | | | 1 | | | | | F | 29 | 0 | 1 | 1 | 0 | 0 | 1 | 0 | 0 | 0 | 0 | | 0 | | 0 | | 0 | | 0 | | 0 | | 0 |
| J339 | 1 |  | | | 1 | | | | | M | 28 | 0 | 1 | 1 | 0 | 0 | 0 | 0 | 1 | 1 | 0 | | 0 | | 0 | | 0 | | 0 | | 0 | | 0 |
| J340 | 0 |  | | | 0 | | | | | M | 20 | 0 | 1 | 1 | 0 | 0 | 0 | 0 | 0 | 0 | 0 | | 0 | | 0 | | 0 | | 0 | | 0 | | 0 |
| J341 | 0 |  | | | 0 | | | | | F | 16 | 0 | 1 | 1 | 0 | 1 | 1 | 1 | 1 | 0 | 0 | | 0 | | 0 | | 0 | | 1 | | 0 | | 0 |
| J342 | 0 |  | | | 0 | | | | | M | 32 | 0 | 1 | 1 | 0 | 1 | 1 | 0 | 0 | 0 | 0 | | 0 | | 0 | | 0 | | 0 | | 0 | | 0 |
| J343 | 1 |  | | | 1 | | | | | F | 31 | 0 | 1 | 0 | 0 | 0 | 0 | 0 | 1 | 0 | 0 | | 1 | | 0 | | 0 | | 0 | | 0 | | 0 |
| J344 | 1 |  | | | 1 | | | | | M | 15 | 0 | 1 | 0 | 1 | 0 | 1 | 0 | 0 | 0 | 0 | | 0 | | 0 | | 0 | | 0 | | 0 | | 0 |
| J345 | 0 |  | | | 0 | | | | | F | 14 | 0 | 1 | 1 | 1 | 0 | 0 | 0 | 0 | 0 | 0 | | 0 | | 0 | | 0 | | 0 | | 0 | | 0 |
| J346 | 0 |  | | | 0 | | | | | F | 49 | 0 | 1 | 1 | 1 | 0 | 0 | 0 | 0 | 1 | 0 | | 0 | | 0 | | 0 | | 1 | | 0 | | 0 |
| J347 | 0 |  | | | 0 | | | | | M | 22 | 0 | 1 | 1 | 1 | 0 | 1 | 0 | 1 | 0 | 0 | | 0 | | 0 | | 0 | | 1 | | 0 | | 0 |
| J348 | 0 |  | | | 0 | | | | | M | 27 | 0 | 1 | 1 | 0 | 1 | 1 | 0 | 0 | 0 | 0 | | 0 | | 0 | | 0 | | 0 | | 0 | | 0 |
| J349 | 1 |  | | | 1 | | | | | F | 22 | 0 | 0 | 1 | 0 | 0 | 1 | 0 | 0 | 0 | 0 | | 1 | | 0 | | 0 | | 0 | | 0 | | 0 |
| J350 | 1 |  | | | 1 | | | | | M | 30 | 0 | 1 | 1 | 0 | 0 | 0 | 0 | 1 | 0 | 0 | | 0 | | 0 | | 0 | | 0 | | 0 | | 0 |
| J351 | 0 |  | | | 0 | | | | | M | 19 | 0 | 1 | 1 | 1 | 1 | 0 | 0 | 1 | 0 | 0 | | 0 | | 0 | | 0 | | 0 | | 0 | | 0 |
| J352 | 0 |  | | | 0 | | | | | M | 18 | 0 | 1 | 1 | 1 | 0 | 1 | 0 | 0 | 0 | 0 | | 0 | | 0 | | 0 | | 0 | | 0 | | 0 |
| J353 | 0 |  | | | 0 | | | | | M | 28 | 0 | 1 | 1 | 1 | 0 | 1 | 0 | 1 | 0 | 1 | | 0 | | 0 | | 0 | | 1 | | 0 | | 0 |
| J354 | 1 |  | | | | | 2 | | | F | 26 | 0 | 1 | 1 | 0 | 0 | 1 | 0 | 0 | 0 | 0 | | 0 | | 0 | | 0 | | 0 | | 0 | | 0 |
| J355 | 1 |  | | | | | 3 | | | M | 21 | 0 | 1 | 1 | 0 | 0 | 0 | 0 | 0 | 0 | 0 | | 0 | | 0 | | 0 | | 0 | | 0 | | 0 |
| J356 | 0 |  | | | | | 0 | | | F | 36 | 0 | 1 | 1 | 0 | 1 | 0 | 0 | 1 | 0 | 0 | | 0 | | 0 | | 0 | | 0 | | 0 | | 0 |
| J357 | 1 |  | | | | | 1 | | | F | 26 | 0 | 1 | 1 | 0 | 0 | 1 | 0 | 0 | 0 | 0 | | 0 | | 0 | | 0 | | 0 | | 0 | | 0 |
| J358 | 1 |  | | | | | 1 | | | M | 24 | 0 | 1 | 0 | 0 | 0 | 0 | 0 | 0 | 0 | 0 | | 0 | | 0 | | 0 | | 0 | | 0 | | 0 |
| J359 | 1 |  | | | | | 2 | | | M | 60 | 1 | 1 | 1 | 0 | 0 | 1 | 0 | 0 | 1 | 0 | | 1 | | 0 | | 0 | | 0 | | 0 | | 0 |
| J360 | 0 |  | | | | | 0 | | | F | 27 | 0 | 1 | 1 | 0 | 1 | 1 | 0 | 1 | 0 | 0 | | 0 | | 0 | | 0 | | 0 | | 0 | | 0 |
| J361 | 1 |  | | | | | 1 | | | F | 22 | 0 | 1 | 1 | 1 | 0 | 0 | 0 | 0 | 0 | 0 | | 0 | | 0 | | 0 | | 0 | | 0 | | 0 |
| J362 | 1 |  | | | | | 1 | | | F | 30 | 0 | 1 | 1 | 0 | 0 | 1 | 0 | 0 | 0 | 0 | | 1 | | 0 | | 0 | | 0 | | 0 | | 0 |
| J363 | 1 |  | | | | | 1 | | | F | 26 | 0 | 1 | 1 | 0 | 0 | 1 | 0 | 0 | 0 | 0 | | 1 | | 0 | | 0 | | 0 | | 0 | | 0 |
| J364 | 0 |  | | | | | 0 | | | M | 30 | 0 | 1 | 1 | 1 | 0 | 1 | 0 | 1 | 0 | 0 | | 0 | | 0 | | 0 | | 1 | | 0 | | 0 |
| J365 | 1 |  | | | | | 1 | | | M | 25 | 0 | 1 | 1 | 1 | 0 | 0 | 0 | 0 | 0 | 0 | | 0 | | 0 | | 0 | | 0 | | 0 | | 0 |
| J366 | 1 |  | | | | | 1 | | | F | 22 | 0 | 1 | 1 | 0 | 0 | 1 | 0 | 1 | 0 | 0 | | 0 | | 0 | | 0 | | 0 | | 0 | | 0 |
